# Artificial Intelligence and Digital Technologies in Orthognathic and Reconstructive Maxillofacial Surgery: Data Availability and Evidence Maturity

**DOI:** 10.3390/dj14090605

**Published:** 2026-09-18

**Authors:** Martín Campuzano-Donoso, Yamilé Dominique Fonseca-Lascano, Paulina Fernanda Galárraga-Taco, Joaquín Alonso Ubidia-Terán, Isaí Alejandro Flores-Reimundo, Ashlee Estephania Insuasti-Veintimilla, Jhon Steven Proaño-Hernández, Jonathan Patricio Zapata-Nuñez, Jairo Jhosue Barrera-Meza, Juan Marcos Parise-Vasco, Claudia Reytor-González

**Affiliations:** 1 Center for Evidence Ecosystems, Implementation Science, and Decision-Making (CIDES), Facultad de Ciencias de la Salud y Bienestar Humano, Universidad Tecnológica Indoamérica, Ambato 180150, Ecuador; martincd01@hotmail.com (M.C.-D.); juanparise@uti.edu.ec (J.M.P.-V.); 2 Facultad de Ciencias Médicas, de la Salud y de la Vida, Escuela de Odontología, Universidad Internacional del Ecuador, Quito 170411, Ecuador; yamilefonsecadl01@gmail.com (Y.D.F.-L.); pauligalarraga@outlook.com (P.F.G.-T.); joaquinubidiateran@gmail.com (J.A.U.-T.); isfloresre@uide.edu.ec (I.A.F.-R.); ashlee.insuasti@gmail.com (A.E.I.-V.); jhproanohe@uide.edu.ec (J.S.P.-H.); jonagato192020@gmail.com (J.P.Z.-N.); jairobarreram2004@hotmail.com (J.J.B.-M.)

**Keywords:** artificial intelligence, orthognathic surgery, maxillofacial surgery, virtual surgical planning, cone-beam computed tomography, augmented reality, robotic surgical procedures, deep learning

## Abstract

Orthognathic and reconstructive maxillofacial surgery addresses severe dentofacial deformities, post-traumatic defects, and defects following oncologic resection. Across the pathway from preoperative imaging and virtual planning to soft-tissue prediction and intraoperative guidance, artificial intelligence and related digital technologies are increasingly being investigated to support decision-making, simulation, and plan transfer. This structured narrative review synthesizes evidence across that surgical pipeline and uses data availability as an organizing framework for interpreting evidence maturity. Predefined searches of PubMed/MEDLINE, Embase, and Scopus identified English-language peer-reviewed articles published from January 2018 to April 2026, supplemented by selected foundational studies. Within the literature reviewed, automated cephalometric and three-dimensional landmark detection has undergone the most extensive quantitative evaluation, supported by several systematic reviews and meta-analyses and by multicenter retrospective evaluations, including one study with independent external test sets. Artificial intelligence-assisted diagnosis, osteotomy planning, and virtual surgical planning show increasing technical capability, including low-millimeter reposition-vector prediction, although external validation and patient-centered outcomes remain limited. Soft-tissue prediction has advanced through deep learning and finite-element modeling, with selected studies reporting comparable geometric accuracy and substantially faster computation. In contrast, intraoperative augmented reality, navigation, and robot-assisted craniomaxillofacial surgery remain supported mainly by small clinical series, cadaveric studies, and preclinical validation. These technologies demonstrate plan-transfer feasibility in selected settings but do not yet establish broad clinical effectiveness or routine superiority over established workflows. Overall, evidence is more mature when datasets are structured, accessible, and standardized, and less mature when paired longitudinal imaging or intraoperative tracking data are sparse. This association should be interpreted as an organizing hypothesis rather than proof of causality.

## 1. Introduction

Orthognathic and reconstructive maxillofacial surgery is used to manage severe dentofacial deformities, post-traumatic defects, and defects following oncologic resection of the facial skeleton. Across these procedures, perioperative care often involves a sequence of measurable tasks, including identification of anatomic landmarks on imaging, design of osteotomies and bony reference points, prediction of postoperative soft-tissue change, and transfer of the surgical plan to the patient. Artificial intelligence (AI), including machine learning, deep learning, computer vision, and generative modeling, has increasingly been investigated across these tasks as part of the broader digitalization of surgery, alongside virtual surgical planning (VSP), robotic assistance, and augmented reality (AR) [1]. The maxillofacial domain is well suited to data-driven investigation because many relevant anatomic structures are captured in three dimensions, lateral cephalograms and cone-beam computed tomography (CBCT) are widely used in clinical practice, and surgical outcomes can often be described using geometric, functional, and aesthetic parameters [1,2].

However, the suitability of this field for AI should not be assumed to be the same across all surgical tasks. Each stage of the orthognathic and reconstructive workflow produces different types of data and varies in how easily those data can be annotated, whether reliable reference standards exist, whether multicenter validation is feasible, and whether technical outputs can be linked to clinically meaningful outcomes [3,4]. For this reason, data availability is used in this review as both a conceptual and methodological lens, rather than simply as a measure of whether images or clinical records exist. In this context, data availability refers to the extent to which task-specific data are structured, sufficiently large, consistently acquired, annotated using reproducible reference standards, representative of diverse clinical populations, and accessible for internal or external validation [3,4]. Evidence maturity refers to the degree to which a technology has moved beyond proof-of-concept development toward external validation, prospective evaluation, workflow integration, and assessment of clinical or patient-centered outcomes [5,6]. This distinction matters because a model may show low numerical error in a controlled dataset but still lack evidence of generalizability, safety, workflow value, or clinical benefit [4,7]. Surgical data science has similarly emphasized that data-driven innovation depends on capturing, organizing, and modeling heterogeneous perioperative data. At the same time, the broader surgical AI literature highlights validation, transparency, human-AI interaction, and prospective evaluation as necessary steps for responsible clinical translation [3,8].

AI applications in orthognathic and oral and maxillofacial surgery have been described across several interconnected domains, including radiographic analysis, diagnosis, VSP, intraoperative guidance, soft-tissue simulation, and outcome evaluation. Reported applications include prediction of surgical need, automated cephalometric analysis, simulation of postoperative facial morphology, and support for surgical planning workflows [1,2]. The level of evidence, however, is uneven. Scoping and systematic reviews have reported variable performance for identifying patients who may require orthognathic surgery and for predicting postoperative soft-tissue change, but these estimates derive from heterogeneous study designs, model architectures, outcome definitions, and validation settings [2,9]. Three-dimensional outcome modeling remains supported mainly by retrospective and single-center studies, while soft-tissue prediction has progressed from traditional biomechanical and statistical approaches toward deep neural networks, PointNet-based models, and transformer-based architectures [9,10]. Across cephalometric landmark-detection studies, automated coordinates were generally compared with study-specific manual reference annotations. When examiner specialty was reported, the annotations were produced most often by orthodontists and occasionally by radiologists, while some studies used established annotated datasets. Accuracy was usually reported as mean radial error, calculated from the Euclidean distance between automated and reference coordinates, or as successful detection rate, the proportion of landmarks within a prespecified distance threshold [11,12,13].

Several recent reviews have examined AI applications in oral and maxillofacial surgery, orthognathic surgery, cephalometric image analysis, three-dimensional outcome modeling, postoperative soft-tissue prediction, and digital transfer workflows [1,2,9,10,14,15,16]. These reviews are valuable because they identify key applications, summarize reported performance, and highlight methodological heterogeneity within specific domains [2,9,10,14]. The present review takes a different approach by using data availability as the organizing framework to examine why the depth and type of validation differ across the surgical pipeline [3,17]. It is not intended to replace systematic reviews, scoping reviews, or meta-analyses, nor does it aim to provide exhaustive evidence ascertainment or pooled estimates. Instead, it synthesizes representative evidence across the perioperative workflow to examine how the type, structure, standardization, and validation of data may influence the translational readiness of AI and related data-driven surgical technologies.

This uneven distribution of evidence suggests that technical performance alone is insufficient to determine clinical readiness. Recurrent limitations include single-center datasets, limited multicenter validation, heterogeneous training and evaluation methods, and variable outcome definitions [1,10,17]. Critical analyses of AI in orthodontic and dental research have similarly noted that many algorithms are trained and evaluated on unicentric datasets, which may increase the risk of bias and limit generalizability. Broader clinical translation is therefore likely to require not only improved model architecture, but also larger and more diverse datasets, standardized data formats, transparent annotation practices, and external validation across populations and clinical settings [17]. Recent multicenter case series of intraoperative AR navigation in craniomaxillofacial (CMF) and head and neck surgery also suggest that selected plan-transfer technologies are being evaluated beyond isolated proof-of-concept settings, although comparative clinical evidence remains limited [18].

**Figure 1:**
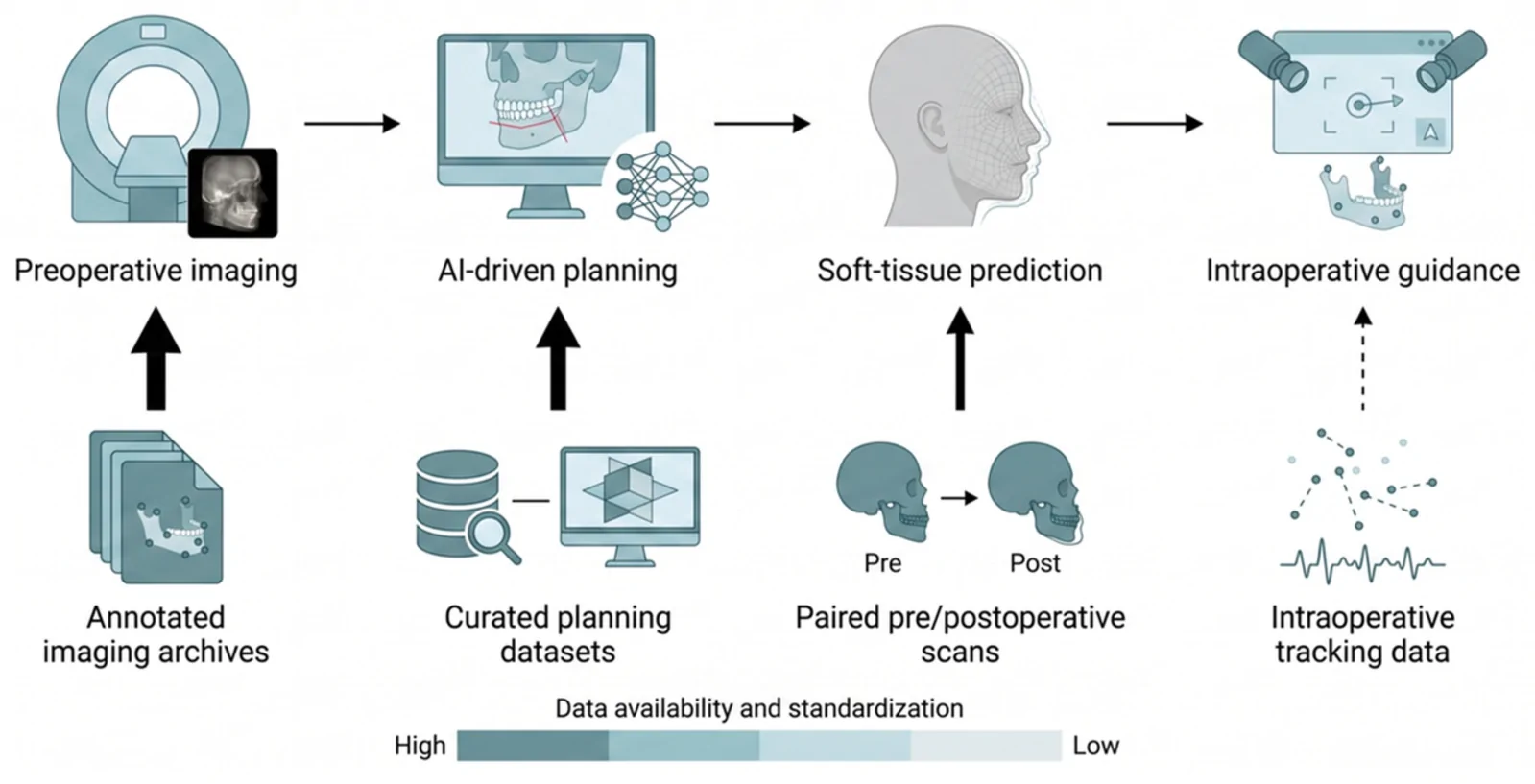
The orthognathic AI pipeline and its associated data resources. Conceptual four-stage pipeline of predictive artificial intelligence and related data-driven digital surgical technologies in orthognathic and reconstructive maxillofacial surgery. Preoperative imaging, AI-assisted planning, soft-tissue outcome prediction, and intraoperative plan transfer are aligned with the types of data that commonly support each stage. Wider arrows indicate relatively more structured and accessible data resources, such as annotated cephalometric and CBCT imaging archives. Narrower or dashed arrows indicate data resources that are typically scarcer or less standardized, including paired preoperative/postoperative imaging and intraoperative registration or tracking data. The figure presents data availability as an organizing framework for interpreting evidence maturity, not as proof of a causal relationship between dataset availability and clinical translation [2,9,17,18,19]. Abbreviations: AI, artificial intelligence.

A less explicitly addressed issue is how differences in data availability across surgical tasks may shape the maturity of AI translation. Automated landmark detection benefits from densely annotated cephalometric and CBCT imaging datasets, standardized anatomic targets, and relatively clear reference standards. Soft-tissue prediction, by contrast, requires paired preoperative and postoperative imaging, which is less abundant and more difficult to standardize. Intraoperative navigation and robotic-assisted workflows depend on registration, tracking, and procedural data that remain comparatively sparse and heterogeneous across institutions. This gradient may help account for why landmark detection has been evaluated more extensively than soft-tissue prediction, AR navigation, and robotic-assisted execution. Figure 1 illustrates this four-stage perioperative pipeline and the relative data resources supporting each stage.

This narrative review uses data availability as an organizing perspective to summarize evidence for predictive AI and related digital surgical technologies across the surgical pipeline, from preoperative imaging and planning to soft-tissue outcome modeling and intraoperative guidance. The review focuses on applications involving geometric prediction, classification, simulation of surgical outcomes, and transfer of surgical plans to the operative field. For clarity, augmented reality, navigation, and robotic-assisted execution are treated as plan-transfer technologies rather than as purely predictive AI applications. The review does not address primarily administrative or non-surgical AI applications, such as workflow optimization, voice-based documentation, or triage chatbots, and it excludes dental implant planning unless directly relevant to reconstructive or craniomaxillofacial surgical workflows. Pediatric craniofacial and cleft-specific applications are discussed only when they inform the broader orthognathic or reconstructive maxillofacial context.

## 2. Materials and Methods

This article was designed as a structured narrative review of predictive AI and related data-driven digital surgical technologies in orthognathic and reconstructive maxillofacial surgery. A literature search using predefined database-specific strategies was used to define the thematic scope of the review, identify representative and clinically relevant evidence across major stages of the surgical workflow, and support a workflow-oriented narrative synthesis. The review was not designed to provide exhaustive evidence ascertainment, a PRISMA flow record, quantitative pooling, or formal certainty-of-evidence assessment. The methodological objective was therefore not to determine a pooled effect estimate for a single intervention, but to map how different types of data and validation strategies support different levels of evidence maturity across the surgical pipeline.

PubMed/MEDLINE, Embase, and Scopus were searched because, together, they provide broad coverage of biomedical, clinical, and interdisciplinary literature relevant to artificial intelligence, computer-assisted surgery, surgical navigation, and craniomaxillofacial technologies. PubMed/MEDLINE was used to develop the core search framework, and the search syntax was adapted for Embase and Scopus according to the indexing structure, controlled vocabulary, and title/abstract search fields available in each database. The search focused on English-language, peer-reviewed articles published between 15 January 2018 and 15 April 2026. Selected pre-2018 articles were also considered when they represented foundational methodological work directly relevant to soft-tissue prediction, finite-element modeling, surgical navigation, or computer-assisted maxillofacial surgery. Web of Science, Cochrane Library, engineering databases, and computer-science conference proceedings were not searched; this restriction was considered when interpreting the breadth and completeness of the evidence.

The search was organized around predefined domains corresponding to the clinical pipeline addressed in the review: cephalometric AI and three-dimensional landmark detection; AI-assisted diagnosis, osteotomy planning, and virtual surgical planning; postoperative soft-tissue outcome prediction; intraoperative navigation, augmented reality, and robotic-assisted craniomaxillofacial surgery; and clinical translation, external validation, generalizability, bias, implementation, regulation, and human oversight. The search framework, domain structure, database-specific syntax for PubMed/MEDLINE, Embase, and Scopus, limits, and evidence-mapping approach are provided in the Supplementary Materials. This domain-based strategy was used to ensure that the review captured both task-specific evidence and cross-cutting translational issues. The first four domains corresponded to clinically recognizable stages of digital orthognathic and reconstructive care, whereas the final translational domain was used to identify literature addressing validation, dataset bias, governance, implementation, and human oversight. In this way, the search strategy directly informed the conceptual framework of the review by linking each surgical stage to the types of data, reference standards, validation methods, and clinical endpoints most commonly available for that stage.

Within the date and language limits described above, sources were considered for narrative discussion when they addressed one or more of the predefined domains and reported information relevant to technical performance, validation, clinical feasibility, or implementation. Evidence types considered included original clinical studies, technical validation studies, systematic reviews, meta-analyses, scoping reviews, narrative reviews, umbrella reviews, cadaveric studies, phantom studies, and translational reports. Database results were reviewed manually by two authors (M.C.-D. and J.M.P.-V.). No automated tools were used for source selection.

Selection of representative sources was guided by the availability of domain-level synthesis and by reported external or prospective validation, comparison with a relevant reference standard or surgical plan, quantitative performance or clinical outcome data, intraoperative feasibility, or implementation barriers. Systematic reviews and meta-analyses were prioritized when they provided relevant domain-level synthesis.

Sources were not selected for detailed discussion when they primarily addressed administrative AI, nonsurgical workflow optimization, voice-based documentation, triage chatbots, or dental implant planning without direct relevance to reconstructive or craniomaxillofacial surgical workflows. Sources were also not emphasized when they lacked sufficient methodological or outcome detail for the cited point or when another source addressed that point more directly.

After selection, sources were organized according to the four clinical stages and their dominant data types, including two-dimensional cephalograms, CBCT or CT volumes, three-dimensional surface data, paired preoperative and postoperative imaging, virtual surgical plans, registration or tracking data, phantom or cadaveric data, and clinical procedural data. Evidence maturity was interpreted according to study design, validation setting, dataset and sample characteristics, reference standard or comparator, reported technical or clinical outcomes, and clinical directness. Full-text sources were used whenever accessible. Abstract-level information was used cautiously and only when sufficient detail was available to support a specific descriptive statement.

The four-stage structure was selected because it reflects the practical sequence through which digital information is generated, transformed, simulated, and transferred in orthognathic and reconstructive maxillofacial surgery. Anatomical landmark identification represents morphometric quantification; diagnostic and surgical planning translates measurements into clinical decisions and VSP workflows; soft-tissue outcome prediction estimates patient-specific facial response; and intraoperative guidance, AR, navigation, and robotic assistance represent plan transfer within the operative field.

Because this article was designed as a narrative review, it did not follow PRISMA methodology, include a formal risk-of-bias assessment, perform quantitative evidence pooling, or apply a formal certainty-of-evidence grading system. Narrative-review transparency principles were used to improve clarity of scope, search reporting, evidence presentation, and cautious interpretation of cited evidence [20]. The interpretation dimensions described above were not combined into a numerical score or formal certainty grade. Numerical estimates were interpreted descriptively because reported metrics varied across tasks, imaging modalities, validation designs, clinical indications, and outcome definitions.

Use of generative artificial intelligence and artificial intelligence-assisted technologies in the writing process: During the preparation and editorial adaptation of this manuscript, OpenAI Codex GPT 5.5 was used solely to support language editing, wording refinement, text condensation, and consistency checks. It was not used to generate or analyze data, determine study eligibility, create figures, or formulate the manuscript’s key arguments or conclusions. The authors reviewed and verified all output and take full responsibility for the content of the manuscript.

## 3. Narrative Synthesis Across Clinical Stages

The evidence was synthesized according to four clinical stages in which predictive AI and related digital technologies may influence orthognathic and reconstructive maxillofacial surgery: anatomical landmark identification, diagnostic and surgical planning support, soft-tissue outcome prediction, and intraoperative transfer of the surgical plan. This structure reflects the clinical sequence of digital surgical care, moving from anatomical quantification to treatment planning, postoperative outcome estimation, and operative execution or plan transfer. This structure also reflects a gradient in data availability. Earlier image-based stages are generally supported by more structured and annotatable data, whereas later simulation and intraoperative stages require longitudinal, procedural, registration, tracking, or achieved-outcome data that are more difficult to standardize and validate across institutions.

Performance metrics differ substantially across these stages and should not be interpreted as directly comparable. Landmark detection studies commonly report mean radial error or agreement with manual reference annotations; planning studies may report reposition-vector error, classification accuracy, or agreement with surgeon-generated plans; soft-tissue prediction studies frequently use surface deviation or qualitative simulation acceptance; and intraoperative guidance studies may report registration error, angular deviation, operative time, or feasibility. For this reason, the evidence is summarized descriptively according to clinical task, data type, validation design, clinical directness, and reported outcome type rather than through numerical comparison alone. Table 1 summarizes representative studies, sample sizes, study designs, reported metrics, and interpretive limitations.

Representative reported metrics, sample sizes, and study designs across selected studies of predictive artificial intelligence and related digital surgical technologies in orthognathic and reconstructive maxillofacial surgery. Metrics are reported as presented in the cited studies and should not be interpreted as directly comparable across tasks, imaging modalities, validation designs, or clinical indications. The table is intended to contextualize the narrative synthesis rather than rank technologies by accuracy or clinical maturity. For interpretive purposes, the evidence can be summarized using four narrative maturity categories: more extensive quantitative validation for 2D and selected 3D landmarking; emerging clinically oriented validation for diagnostic, planning, and VSP-related models; methodological or early translational evidence for soft-tissue prediction; and preclinical or feasibility-dominant evidence for AR navigation and robotic-assisted execution. These categories are interpretive descriptors rather than formal grades of certainty. Abbreviations: AI, artificial intelligence; AR, augmented reality; AUC, area under the curve; CBCT, cone-beam computed tomography; CI, confidence interval; CMF, craniomaxillofacial; CT, computed tomography; DL, deep learning; FEM, finite-element method; ML, machine learning; SCT, spiral computed tomography; SD, standard deviation; VSP, virtual surgical planning.

### 3.1. Automated Cephalometric and Three-Dimensional Landmark Detection

Cephalometric analysis provides the morphometric basis for orthodontic and craniomaxillofacial surgical planning. Automated landmark detection is one of the most extensively studied AI applications in this field because it addresses a repetitive, image-based task with anatomically defined targets. Within the literature reviewed, this domain has a broader quantitative literature than tasks that require surgical-plan generation, postoperative soft-tissue modeling, or intraoperative plan transfer. Several features may contribute to this pattern. Lateral cephalograms and CBCT scans are routinely acquired, landmarks are generally defined according to established anatomical conventions, manual reference annotations can be repeated and audited, and performance can be evaluated using spatial metrics such as mean radial error and successful detection rate [11,34]. Together, these features create a more structured data environment than is typically available for surgical-plan generation, individualized soft-tissue response prediction, or intraoperative execution [9,19].

A systematic review and meta-analysis included 34 studies of AI-driven cephalometric landmarking, of which 27 evaluated two-dimensional lateral cephalograms and seven evaluated three-dimensional CBCT volumes [11]. Fourteen of the two-dimensional studies, contributing 21 estimates, were included in the quantitative synthesis. The main analysis reported a pooled mean radial error of 1.43 mm, with a 95% confidence interval of 0.95 to 1.91 mm [11]. This estimate falls below the 2 mm threshold commonly reported in cephalometric AI studies, but that threshold is a reporting convention rather than a universal criterion of clinical acceptability. Its relevance depends on the landmark, imaging modality, reference construction, and clinical decision being supported [11,34]. Automated landmarking was also reported to require less than one minute per case, compared with approximately fifteen minutes for manual tracing [11]. For three-dimensional imaging, errors ranged from 1.0 to 5.8 mm in the same review, but heterogeneity prevented meta-analysis [11].

A systematic review and meta-analysis of 40 studies reported pooled successful detection rates of 79% within 2 mm and 90% within 3 mm of manual reference annotations, but the certainty of evidence was rated very low [13]. A separate review of 15 three-dimensional studies included 11 in the meta-analysis and reported a pooled mean error of 2.44 mm, with a 95% confidence interval of 1.83 to 3.05 mm and I^2^ of 98.13%, indicating substantial heterogeneity [12]. Convolutional neural networks, You Only Look Once detectors, Bayesian convolutional networks, stacked-hourglass networks, and multistage models have all been used, but differences in datasets and reference construction limit direct comparisons [11,12,35].

Reference-standard construction varied across studies. Across the 40 studies reviewed by Mesquita et al., one to five examiners were involved when reported, most often orthodontists and occasionally radiologists, while several studies did not describe examiner specialty or reliability testing [13]. In the widely used IEEE ISBI dataset, one junior and one senior orthodontic specialist independently annotated 19 landmarks, and the mean of their coordinates was used as the reference [11]. A common comparison was the Euclidean distance between corresponding automated and reference coordinates in two- or three-dimensional space. Mean radial error summarizes these distances, whereas successful detection rate reports the proportion within a specified threshold [11,12,13].

More recent work has begun to address dataset heterogeneity. Sahlsten et al. evaluated 46 landmarks in 309 CBCT scans obtained from two centers, three imaging devices, and Finnish and Thai cohorts [36]. The scans were pooled before division into training, validation, and test sets, and each scan received one manual annotation. The study therefore broadened multicenter, multiethnic, and multidevice evaluation, but it did not test between-center generalizability using an independent external dataset [36].

These estimates should be interpreted cautiously. An umbrella review noted that AI systems do not identify all landmarks with equal accuracy and that the commonly used 2 mm cutoff may not be equally meaningful for every anatomical reference point or clinical application [34]. A separate multicenter retrospective study used independent external test sets [21]. Its lightweight three-dimensional U-Net was developed using 480 spiral computed tomography scans and 240 CBCT scans and evaluated on external sets of 320 spiral computed tomography scans and 150 CBCT scans. Mean radial errors remained below 1.3 mm overall and below 1.4 mm in cases with malocclusion, missing dental landmarks, or metal artifacts [21]. Performance was similar across internal and external datasets. Within SCT, bone landmarks were more precise than dental landmarks, while CBCT dental landmarks were more precise than CBCT bone landmarks. Prospective clinical validation is still required [21].

The clinical importance of landmarking error depends on how the landmark is used. A small millimetric error may have limited practical impact when a landmark is used only for descriptive cephalometric assessment. However, the same degree of error may be more consequential when the landmark contributes to sagittal jaw classification, vertical facial assessment, occlusal-plane orientation, asymmetry analysis, or surgical-movement quantification [11,34]. Global mean error can also hide clinically important differences in landmark-specific performance, particularly for dental landmarks affected by imaging artifacts or soft-tissue landmarks with less reproducible anatomical boundaries [12,21]. As landmarking models move closer to clinical use, recent AI reporting and evaluation frameworks may provide useful methodological guidance. These include DECIDE-AI for early clinical evaluation, TRIPOD + AI for prediction models, PROBAST + AI for risk of bias and applicability, and STARD-AI for diagnostic accuracy studies [6,8,37,38]. Earlier non-AI tools, including STARD 2015, TRIPOD, and PROBAST, also remain important foundations for transparency in diagnostic and prediction-model research [39,40,41].

Overall, automated cephalometric and three-dimensional landmark detection has a comparatively more developed quantitative validation base within the literature reviewed. This assessment reflects the availability of several systematic reviews and meta-analyses, an umbrella review of 11 systematic reviews, and multicenter retrospective evaluations, including one study with independent external test sets [11,12,13,21,34,36]. By comparison, AI-assisted planning and soft-tissue prediction remain characterized by small, heterogeneous, and largely retrospective clinical studies, while intraoperative AR and robotic applications are supported mainly by small case series, cadaveric studies, phantom experiments, and other preclinical evaluations [14,18,28,29,30,31,32,33,42,43,44]. This comparison refers to the presence of repeated quantitative synthesis and multicenter evaluation, not to high certainty of evidence or clinical readiness. Landmarking evidence still shows frequent risk of bias, substantial heterogeneity in three-dimensional estimates, variable landmark-specific performance, and limited prospective evidence of downstream clinical benefit [11,12,13,34].

In relation to the central hypothesis of this review, landmark detection provides one of the clearest examples of how a structured and annotatable data environment can support repeated quantitative evaluation [11,12,21,36]. The next step in evidence maturity is not simply to further reduce mean radial error, but to determine whether automated landmarking improves diagnostic consistency, planning efficiency, clinician workload, reproducibility across populations, and downstream surgical decision-making.

### 3.2. AI-Assisted Diagnosis, Osteotomy Planning, and Virtual Surgical Planning

Beyond landmark detection, AI has been applied to more complex diagnostic and planning tasks, including classification of dentofacial deformities, prediction of surgical need, estimation of reposition vectors, and support for osteotomy design. These applications require more than point localization. They involve the interpretation of skeletal relationships, treatment objectives, surgeon preferences, patient-specific anatomy, and institutional planning protocols. As a result, the evidence base is less uniform than that for automated cephalometric landmarking. Whereas landmark detection can be evaluated against relatively standardized anatomical targets, diagnostic and planning models must approximate higher-level clinical reasoning, making validation more dependent on case mix, treatment philosophy, surgical protocol, and the definition of an acceptable plan. For this reason, AI-assisted planning should be divided conceptually into at least three related but distinct tasks: diagnostic support, generation or ranking of possible surgical plans, and prediction of skeletal movement or reposition vectors [2,22]. A model that performs well in one of these areas should not be assumed to be clinically mature in the others, because each task has different data requirements, clinical risks, and validation endpoints.

Systematic reviews describe a small and heterogeneous evidence base for AI-assisted treatment planning and outcome prediction. Salazar et al. included eight clinical studies, seven retrospective and one randomized, and did not perform a meta-analysis because of methodological inconsistency [14]. Sankar et al. included 14 studies using three-dimensional imaging and likewise did not pool the results because of heterogeneity and incomplete reporting. That review characterized most models as proof-of-concept and called for prospective multicenter validation [42]. In a five-domain scoping review, AI models related to osteotomy planning and bony reference-point placement reported errors of 3.99 to 4.73 mm [2]. These values should not be compared directly with landmark-detection errors because planning models address different tasks, data structures, and clinical endpoints. Overall, the current validation structure limits confidence that models trained on local planning preferences will generalize across surgical teams, populations, or treatment philosophies.

Recent planning studies illustrate both the technical potential of AI-assisted workflows and the need for cautious interpretation. A Transformer-based regression network, referred to as a VSP transformer, was developed using 383 retrospective samples and clinically tested on 49 prospectively collected samples. The model predicted ten landmark-based reposition vectors with mean absolute errors of 1.41 mm in the validation set and 1.34 mm in the clinical test set. Permutation-importance analysis was reported to be consistent with clinical knowledge and experience [22]. These findings support the potential of AI for planning assistance, although they should be interpreted in relation to the study design, dataset, and need for broader external validation.

Reposition-vector prediction is clinically appealing because it turns part of the planning process into a measurable geometric task [22]. However, a predicted vector should not be treated as a complete surgical plan. Full planning also requires assessment of occlusion, dental decompensation, airway considerations, temporomandibular joint status, facial esthetics, periodontal limitations, stability, relapse risk, patient expectations, and the surgeon’s preferred execution strategy [45,46].

Other approaches have combined diagnostic classification with surgical plan generation. A machine learning-based decision-support system trained on spiral computed tomography data from 574 patients with dento-maxillofacial deformities reported diagnostic success rates above 90% for six deformity categories, with maxillary overdevelopment as the exception at 89.27%, and AUC values above 0.88 for all diagnostic types. In the same study, an adaptive artificial bee colony algorithm generated surgical plans that were evaluated in a 50-patient cohort. The median surgical-plan score was 9 and improved after human–computer interaction, with no statistically significant difference reported between the actual and AI-generated planning groups [23]. These findings suggest that AI may help with diagnostic stratification and preliminary surgical planning. However, they should not be interpreted as evidence that algorithmic systems can replace surgeon-led treatment planning. The human–computer interaction component is especially important because it suggests that model output may be most useful as a draft, comparison tool, or optimization aid rather than as an autonomous final decision. In clinical practice, an acceptable plan is not defined only by geometric similarity to a training-label plan. It also depends on surgical feasibility, biological reasonableness, stability, esthetic acceptability, and alignment with the overall treatment sequence [23,42].

Diagnostic prediction has also been explored using cephalometric and photographic data. Ten machine learning models were evaluated on 920 lateral radiographs from patients previously treated with orthodontics alone or combined orthodontic–orthognathic surgery, including 558 Class II and 362 Class III patients. In the testing dataset, the combined 10-model approach reported accuracy, F1 score, and AUC values of 0.707, 0.706, and 0.791 for the entire sample; 0.759, 0.758, and 0.824 for Class II patients; and 0.822, 0.807, and 0.890 for Class III patients [47]. This subgroup pattern is clinically relevant because orthognathic diagnosis is not a single binary task. It depends on skeletal class, asymmetry, dental compensation, soft-tissue profile, functional considerations, and treatment objectives.

AI-based surgical-need classification has similarly been investigated using facial photographs and cephalograms, with the goal of assisting diagnostic screening and early decision-making in patients with dentofacial deformities. A VGG19 convolutional neural network trained on frontal and lateral facial photographs from 822 patients classified 366 of 410 test cases correctly, yielding an accuracy of 89.3%, precision of 0.912, recall of 0.867, and F1 score of 0.889 for identifying profiles requiring orthognathic surgery [48]. A separate deep learning model based on lateral cephalograms from 840 patients with Class II, Class III, and facial asymmetry patterns demonstrated high accuracy, sensitivity, and specificity for predicting the need for orthognathic surgery [49]. These tools may help standardize early assessment and support clinicians during initial evaluation, but they should not be viewed as replacements for comprehensive clinical diagnosis. Their performance may depend on population characteristics, imaging protocols, skeletal-pattern distribution, and local thresholds for recommending surgery. Surgical-need prediction is especially vulnerable to institutional bias because the label “surgery” often reflects previous treatment decisions rather than an objective biological ground truth [47,48,49]. Without validation across different patient groups, referral patterns, and treatment philosophies, models trained on these labels may reproduce local decision-making habits rather than provide broadly generalizable clinical guidance [7,17].

Comparative studies are particularly important because they evaluate whether AI models offer meaningful advantages over established statistical and planning methods. In a cohort of 705 surgical-orthodontic patients, a TabNet deep neural network was compared with multivariate multiple linear regression and partial least squares for predicting 64 Cartesian coordinate variables from 32 postoperative soft-tissue landmarks. Partial least squares were more accurate for 16 landmarks above the upper lip, whereas AI outperformed the conventional models for six landmarks located along the lower mandibular border and neck region. The remaining ten landmarks showed no significant difference between AI and partial least squares [50]. This finding is important because it challenges the assumption that deep learning is automatically superior to conventional modeling. Instead, model performance appears to depend on anatomical variability, data structure, interpretability, and the specific prediction target. Hybrid or clinician-supervised approaches may therefore be more useful than purely automated planning in some contexts.

Generative approaches are beginning to expand the scope of orthognathic planning beyond classification or movement-vector prediction. GPOSC-Net combines graph neural networks for cephalometric landmark prediction with a latent diffusion model capable of synthesizing postoperative lateral cephalograms from preoperative data [51]. The authors validated the model using diverse patient datasets, a visual Turing test, and simulation studies, and reported accurate landmark prediction with high-fidelity synthesized postoperative cephalogram images [51]. Methodologically, this reflects a shift from conventional predictive models toward generative systems that combine anatomical prediction with clinical visualization. However, clinical usefulness still depends on showing that synthesized images improve decision-making, patient communication, plan selection, or postoperative outcomes, rather than simply appearing visually realistic. For generative systems, visual plausibility should be distinguished from anatomical validity and clinical actionability [8,51]. A generated postoperative image may look realistic while still failing to capture occlusal constraints, skeletal stability, condylar seating, airway change, soft-tissue biomechanics, or the limits of surgically achievable movement.

Commercial AI-assisted planning tools require particular caution because their performance may differ substantially from results reported in experimental model-development settings. Four commercially available deep learning-based cephalometric programs were compared with three-dimensional CT reference standards in orthognathic surgery candidates [24]. In that prospective comparative study, 50 CT images were analyzed. ANB angle did not differ significantly between methods, whereas SNA, SNB, Wits appraisal, *Y*-axis angle, and facial-height ratios showed significant discrepancies compared with the three-dimensional reference standard [24]. These findings apply to the evaluated tools and measurement categories. They should not be generalized to all commercial AI systems. Independent validation and clinician oversight remain necessary before such tools can be relied on for routine orthognathic planning. Commercial availability should therefore be interpreted as deployment status, not as evidence maturity. Independent validation should ideally include transparent datasets, clinically relevant subgroups, comparison with expert planning, evaluation of error propagation, and assessment of how software changes clinician decisions or workflow efficiency [6,24].

Virtual surgical planning provides the clinical infrastructure within which many AI-assisted planning tools are being developed. VSP is widely used in specialist orthognathic and reconstructive workflows and serves as a reference framework against which AI-based, navigation-based, or robotic enhancements are frequently evaluated. A meta-analysis of eight articles from five randomized clinical trials, including 199 patients, reported that VSP and traditional planning showed similar hard-tissue accuracy in the sagittal plane, while VSP was more accurate in selected anterior maxillary reference areas and showed greater precision for sagittal soft-tissue prediction. VSP required more software-planning time, but reduced time across the overall preoperative process and could reduce operative time when paired with accurate computer-aided splints [45]. This suggests that the value of VSP should be assessed not only by geometric accuracy, but also by plan transfer, communication, workflow integration, and compatibility with patient-specific guides or emerging AI-assisted systems. A later narrative review also supported the role of VSP, three-dimensional printing, and patient-specific guides in complex or asymmetric cases, although claims about predictability, error reduction, and intraoperative optimization remain dependent on study design and evaluated outcomes [46]. In the context of this review, VSP is also important because it provides one of the few relatively structured planning-data environments available for AI development [22,45]. However, VSP datasets are still less standardized than landmark datasets because they encode decisions made by specific surgical teams using local planning protocols, software tools, splint-design strategies, occlusal goals, and tolerance thresholds [10,42]. Additional VSP-focused reviews have also emphasized that digital planning and digital transfer should be evaluated not only for accuracy but also for efficiency, reproducibility, and implementation context [16,52,53].

Recent bibliometric evidence further supports the increasing research activity around AI-assisted applications in oral and maxillofacial surgery, including orthognathic surgery. In an analysis of 5267 publications, Huang et al. identified a research base dominated by AI-driven medical image analysis, with a shift from traditional machine learning toward deep learning and transformer-based models [54]. Orthognathic surgery was also identified as one of the areas receiving growing attention within AI-driven oral and maxillofacial surgery. However, bibliometric growth should not be interpreted as evidence of clinical maturity. Publication volume may reflect technological interest, methodological development, and expanding imaging-based research rather than routine clinical implementation.

Taken together, AI-assisted diagnosis, osteotomy planning, and VSP-related studies show a field moving from technical feasibility toward clinically oriented validation, but the transition remains incomplete. Larger datasets, prospective testing, and plan-comparison studies are emerging, yet many models are still evaluated within single institutions and through geometric or classification endpoints. At present, the most defensible role of AI is not to replace surgical planning, but to support structured diagnosis, generate preliminary planning options, assist visualization, and reduce selected workflow burdens while leaving indication, treatment objectives, plan approval, and operative responsibility under clinician control.

### 3.3. Soft-Tissue Outcome Prediction

Prediction of postoperative facial soft-tissue changes represents one of the most clinically relevant and technically complex applications of AI in orthognathic surgery. Unlike cephalometric landmark detection, soft-tissue outcome prediction requires modeling the variable interaction between skeletal displacement and facial soft-tissue deformation. These changes may be influenced by patient-specific factors such as anatomy, magnitude and direction of surgical movement, tissue thickness, muscular anatomy, age, sex, and individual biological response. As a result, reproducible and clinically reliable prediction remains difficult. Although deep learning techniques have increasingly entered this field, finite-element modeling (FEM) continues to provide an important biomechanical reference framework rather than an outdated comparator. This distinction matters because soft-tissue prediction is not only a geometric task, but also a biological and biomechanical problem in which individual tissue behavior may limit the precision of purely data-driven models.

The complexity of soft-tissue prediction arises from the fact that the visible facial result is not a direct linear translation of skeletal movement. Similar maxillary, mandibular, or chin movements may produce different facial responses depending on soft-tissue thickness, lip posture, muscle tone, scar tissue, edema resolution, facial asymmetry, age-related elasticity, and the time interval at which the postoperative image is acquired [9,55]. The reference standard is therefore more difficult to define than in landmark detection because postoperative appearance may vary with swelling, orthodontic finishing, weight change, and facial expression during image acquisition [43,56].

Soft-tissue prediction methods can be broadly divided into traditional biomechanical or statistical approaches and AI-based approaches [9]. Traditional methods include soft-to-hard tissue displacement ratios, multiple linear regression, biomechanical simulation, finite-element methods, mass-spring and mass-tensor models, photographic morphing, and finite-difference methods. AI-based approaches include deep neural networks, PointNet, PointNet++, PointConv, transformer-based architectures, and hybrid machine-learning/FEM models [9]. In a scoping review, reported success rates for AI-based soft-tissue prediction ranged from 64.3% to 100% [2]. This range should be interpreted as evidence of substantial heterogeneity rather than as a stable estimate of clinical accuracy, because the included studies differed in model architecture, validation strategy, sample size, outcome definition, and anatomical region evaluated. Recent reviews focused on AI-based orthognathic prediction emphasize that reported performance is difficult to compare across studies because models use different imaging inputs, facial regions, outcome metrics, and validation procedures [42,57]. This heterogeneity supports the need for task-specific reporting and validation rather than cross-domain comparisons based only on surface deviation or percentage accuracy [6,58].

Finite-element methods remain an important benchmark for interpreting newer deep learning systems. A three-stage FEM approach for double-jaw surgery incorporated tissue sliding between the gums and teeth as well as correction of soft-tissue–bone mapping errors [25]. In 40 retrospectively evaluated patients, the model demonstrated an overall facial surface deviation of 1.1 ± 0.3 mm, with upper- and lower-lip prediction errors of 1.2 ± 0.7 mm and 1.5 ± 0.7 mm, respectively. Quantitative distance errors between predicted and actual surfaces were below 1.5 mm for the entire face and evaluated subregions. Expert qualitative evaluation showed a 46% improvement in acceptance rate compared with traditional FEM simulation, and more than 80% of results generated by the improved approach were considered acceptable [25]. These findings support the continued relevance of biomechanical modeling, although the retrospective design and limited sample size should be considered when interpreting clinical applicability.

The conceptual strength of FEM is that it can explicitly represent anatomical structures, material properties, contact relationships, and boundary conditions. This is particularly relevant in regions such as the lips and perioral tissues, where sliding, stretching, and muscle-related deformation may not be adequately represented by simple displacement ratios [25,59]. The limitation is that patient-specific FEM can be computationally demanding and may require assumptions about tissue properties that are difficult to measure directly in routine clinical practice [60,61].

Probabilistic and anatomically refined FEM approaches have attempted to address some of the uncertainty inherent in individualized soft-tissue prediction. A probabilistic FEM model was developed and validated on eight patients who underwent maxillary repositioning and had preoperative and postoperative CBCT scans [60]. Rather than producing a single deterministic result, the model generated possible outcome ranges. Predictions involving the nose and upper lip included the true postoperative position, whereas the cheek and lower-lip regions tended to be underestimated [60]. The small cohort size limits generalizability, but the probabilistic framework is conceptually relevant because it reflects the uncertainty of patient-specific soft-tissue response. Subsequent FEM refinements incorporated a more realistic lip-sliding mechanism within an incremental simulation framework validated on 35 retrospective clinical datasets, demonstrating improved prediction accuracy in the lip region compared with previous FEM variants [59]. Together, these studies suggest that FEM remains useful not only as a comparator for deep learning models, but also as a platform for modeling anatomical constraints and uncertainty.

Clinical validation studies also show that accuracy should be assessed by facial region rather than by a single global surface value. Yamashita et al. evaluated three-dimensional soft-tissue profile prediction in orthognathic surgery, while Awad et al. examined prediction accuracy according to facial aesthetic units [55,56]. Quantitative and qualitative validation may be complementary because numerical surface deviation does not always reflect whether a simulation is acceptable for planning or patient communication [62].

To address the computational demands associated with FEM, several deep learning approaches have been proposed to simplify workflows and reduce processing time while attempting to preserve geometric accuracy. Biomechanics-informed point-cloud models represent one strategy for combining anatomical structure with data-driven prediction. A PointNet++ model using point-cloud data and explicit boundary-type information was developed as a proof-of-concept approach for fast prediction of soft-tissue deformation [63]. This work primarily demonstrated technical feasibility rather than definitive clinical superiority.

ACMT-Net, an Attentive Correspondence-assisted Movement Transformation architecture, was designed to model the relationship between skeletal movement and postoperative soft-tissue change through a point-to-point attentive correspondence matrix [27]. The model incorporated self-supervised pre-training with k-nearest-neighbor clustering and reported facial-change prediction accuracy comparable to a FEM-based method, with significantly improved computational efficiency [27]. FSC-Net, a facial shape-change prediction network based on distance-guided shape and local-point constraint losses, achieved a 15-fold speedup while maintaining accuracy comparable to a state-of-the-art FEM method [26]. These findings support the potential of deep learning to accelerate soft-tissue simulation workflows, but they do not eliminate the need for validation in larger and clinically heterogeneous populations. The conceptual advantage of deep learning is that it can learn nonlinear skeletal-soft-tissue relationships from paired data without requiring all biomechanical assumptions to be specified manually. However, this advantage becomes clinically meaningful only when the training data adequately represent the range of deformities, surgical movements, imaging protocols, and postoperative outcomes encountered in practice [42,43].

Other deep learning models have focused on geometric correspondence and patient-specific reference anatomy. A three-dimensional point-cloud network estimated patient-specific reference bony surfaces using synthetic deformation strategies and demonstrated clinically acceptable reference-shape estimation in comparison with a supervised point-cloud method [64]. Prospective and multimodal investigations remain relatively limited but are important for improving anatomical realism. In one prospective study, eight patients underwent preoperative CBCT, planar radiographs, and MRI to construct patient-specific FEM models incorporating hard tissue, skin, and muscle anatomy [61]. The homogeneous model was compared with postoperative CBCT-based simulation for validation, and the quantitative distance error between the homogeneous model and actual patient surfaces in the midface was 0.55 mm, with a standard deviation of 2.9 mm [61]. Although the cohort was small, the study illustrates how multimodal imaging may enrich soft-tissue prediction models beyond surface geometry alone. Recent deep learning approaches have also expanded beyond conventional CT- or CBCT-based workflows. Berends et al. developed a real-time soft-tissue prediction method based on three-dimensional photographs from 458 orthognathic surgery patients [65]. Bao et al. proposed a bidirectional three-dimensional deep learning framework for facial and skeletal transformations, reflecting a broader shift toward models that learn subject-specific relationships between facial surface morphology and skeletal anatomy [66].

Across both FEM and deep learning methodologies, several recurring limitations remain evident. These include small sample sizes, limited external validation, heterogeneous outcome metrics, insufficient subgroup representation, and variability in imaging protocols. A scoping review of AI-based prediction of postoperative facial changes emphasized small cohorts and lack of external validation as major limitations [43]. Similarly, a systematic review focused on non-syndromic facial asymmetry highlighted the need to interpret virtual soft-tissue prediction in relation to anatomical region, software workflow, and validation method [44]. This regional variability is clinically important because lower-face prediction may be more affected by soft-tissue thickness, muscular function, mandibular movement, and individual biological response than by skeletal displacement alone. A central data limitation is the need for paired longitudinal imaging. Robust soft-tissue prediction requires preoperative imaging, accurate skeletal-movement data, and postoperative imaging acquired at a clinically meaningful and standardized time point [9,43]. Without consistent follow-up intervals, models may learn a mixture of surgical effect, postoperative swelling, orthodontic finishing, facial expression variation, and measurement noise rather than stable treatment outcome [55,62].

The central translational barrier for soft-tissue prediction is therefore not only algorithmic performance, but also the availability of clinically meaningful longitudinal data. Unlike landmark detection, where annotated imaging datasets are more accessible and anatomically standardized, soft-tissue prediction requires paired preoperative and postoperative imaging, reliable surgical movement data, and follow-up intervals that capture the postoperative state being modeled. Deep learning may reduce computational burden and improve workflow efficiency in selected scenarios, but broader clinical use will depend on external validation, standardized reporting, and better access to paired imaging datasets. Current evidence supports these technologies primarily as adjuncts for planning and patient communication rather than as definitive predictors of individualized postoperative facial appearance.

### 3.4. Intraoperative Guidance, Augmented Reality, and Robotic-Assisted Plan Transfer

The final stage in translating a virtual surgical plan into a clinical outcome involves transferring the preoperative design to the patient during surgery. In this context, the primary challenge extends beyond prediction and includes accurate registration, tracking, visualization, and surgical execution. Consequently, intraoperative AR and robotic-assisted systems should be understood as complementary digital surgical technologies rather than purely predictive AI tools. Their relevance lies in how AI-assisted or VSP-derived plans are implemented within the operative field. This stage is therefore less dependent on prediction alone and more dependent on the fidelity of plan transfer, the stability of image-to-patient registration, and the surgeon’s ability to verify and adapt the digital plan intraoperatively.

This distinction is important because plan transfer generates a different type of evidence problem from preoperative image analysis. Landmarking and many planning models can be evaluated using static images, annotated reference points, or virtual-plan comparisons, whereas intraoperative technologies must maintain spatial accuracy under surgical conditions that include patient positioning, tissue displacement, limited access, blood, instrument occlusion, lighting variation, and workflow interruptions [19,67].

Image-to-patient registration remains a major technical challenge in craniomaxillofacial surgery, particularly in mandibular procedures. A systematic review of 81 articles identified four principal registration approaches: point-based, surface-based, hybrid, and computer vision-based methods [19]. Mandibular mobility represents a specific limitation because the intraoperative mandibular position may differ from its position during preoperative imaging, requiring either fixed-position imaging protocols or active tracking of mandibular movement [19]. Craniofacial navigation systems have also been affected by calibration inaccuracies, registration errors, and soft-tissue deformation, even in relatively mature navigation platforms [67]. These limitations are clinically significant because intraoperative guidance systems rely on precise alignment between virtual anatomy and the operative field. If this alignment is unstable, even a well-designed virtual plan may be transferred inaccurately during surgery.

Registration error can propagate through the entire plan-transfer chain, including segmentation, virtual model generation, patient-to-image registration, instrument tracking, visual overlay, guide positioning, osteotomy execution, and verification of the achieved skeletal position [19,67]. Robust validation would ideally require synchronized preoperative imaging, intraoperative registration logs, tracking data, instrument trajectories, timestamps, surgeon interactions, intraoperative video or AR overlays, achieved postoperative anatomy, and clinically meaningful outcomes [18,68]. These data are difficult to capture consistently across institutions because operative workflows, tracking systems, calibration methods, hardware platforms, anatomical indications, and surgeon behavior vary considerably [18,19].

Beyond registration challenges, phantom and cadaveric investigations have provided preliminary technical validation for AR-assisted navigation systems. An optical see-through AR navigation platform for mandibular distraction osteogenesis was evaluated using three-dimensional printed phantom mandibles [30]. In a paired comparison involving 40 distractors, AR guidance reduced mean angular error by 61.6% and displacement error by 57% compared with free-hand positioning [30]. Although these findings support technical feasibility, phantom models cannot fully reproduce the complexity of clinical surgery, including soft-tissue movement, intraoperative bleeding, restricted visualization, and anatomical changes occurring during procedures. Phantom-based accuracy should therefore be interpreted as a technical benchmark rather than direct evidence of clinical effectiveness. Recent technical studies continue to show the potential of AR visualization while illustrating the preclinical-to-clinical gap. A portable AR surgical navigation system for maxillofacial surgery demonstrated an overall overlap error of 0.53 +/− 0.21 mm in a skull-model accuracy study [69]. A comparative AR navigation study in orthognathic surgery also supported the feasibility of AR-based guidance, while emphasizing that translation depends on ergonomics, registration workflow, and procedural integration [70].

Clinical AR investigations remain relatively limited and are predominantly composed of small retrospective or feasibility-oriented studies. A retrospective multicenter case series across four tertiary academic centers evaluated a Microsoft HoloLens 2-based AR platform in 33 craniomaxillofacial and head and neck procedures [18]. Reported applications included instrument tracking during midface osteotomies, zygomaticomaxillary complex fracture reduction, mandibular tracking in orthognathic surgery, AR-assisted cutting guides for fibular reconstruction, and integration of real-time infrared imaging for vascular identification [18]. This experience suggests that AR can be deployed across selected CMF and head and neck workflows, but the retrospective design, heterogeneous indications, and variable outcome measures limit conclusions about comparative accuracy, safety, or clinical superiority. The clinical evidence should therefore be separated from experimental and cadaveric evidence. Clinical series demonstrate that AR systems can be brought into the operating room and used across selected indications, but they frequently combine heterogeneous procedures, small sample sizes, and feasibility-oriented endpoints [18,71]. Phantom and cadaveric studies often provide cleaner accuracy measurements, but they are indirect for patient-centered outcomes because they do not reproduce the full biological and workflow complexity of live surgery [29,30].

Additional studies have reported indication-specific accuracy outcomes. In Le Fort I osteotomy, markerless AR-assisted guidance in 6 patients produced a maxillary-position standard deviation of 0.81 mm, with 81.0% of measurements within 1 mm when postoperative reconstructed CT images were compared with VSP outcomes [28]. Markerless AR guidance using HoloLens 2 has also been described in 3 mandibular and maxillary oncologic resections, where the technique was feasible but improvements in tracking stability remained necessary [71]. In a human cadaver study after total maxillectomy, AR-navigated placement of 20 zygomatic implants in 5 skulls produced a mean entry-point deviation of 2.43 ± 1.33 mm and a three-dimensional angular deviation of 5.80 ± 4.12° [29]. Conventional surgical guides demonstrated superior entry-point and angular accuracy, although no significant differences were observed for exit-point positioning [29]. In zygomaticomaxillary complex fractures, a randomized comparison of 10 patients reported smaller fracture-point error and shorter reduction time with AR navigation than with conventional optical navigation, without differences in operative duration, blood loss, or complications [72]. Taken together, these studies suggest that AR may improve visualization and selected accuracy parameters, but performance remains highly dependent on indication, registration method, tracking stability, and comparator technology.

Reconstructive and oncologic applications show a similar pattern: promising technical feasibility, but limited comparative clinical evidence. Tablet- and HoloLens-based AR systems for fibular flap skin paddle harvesting using three-dimensional printed phantoms reported registration errors ranging from 1 to 5 mm [73]. In simulated mandibular reconstruction, AR-guided osteotomies using HoloLens 2 were feasible, with reported deviations of 4.1° ± 2.6° and 2.0 ± 1.1 mm in the first session, and 3.1° ± 2.3° and 2.3 ± 1.4 mm in the second session [74]. Mixed-reality intraoperative guidance in congenital craniofacial surgery has also been described as a useful adjunctive tool, although not a replacement for conventional cutting guides until more robust registration methods become available [75]. The most technically integrated approaches have combined AR visualization with robotic positioning or optical tracking. In a cadaveric mandibular reconstruction model, AR guidance alone produced mandibular osteotomy errors of 1.61 ± 0.62 mm and fibular osteotomy errors of 1.08 ± 0.28 mm. These values improved to 1.47 ± 0.46 mm and 0.98 ± 0.24 mm, respectively, after integration with robotic positioning systems [33]. This suggests that hybrid AR–robotic workflows may reduce transfer error, but current evidence remains preclinical.

Robotic-assisted craniomaxillofacial surgery remains less mature than AR visualization technologies and considerably less consolidated than robotic systems currently used in implant dentistry. Preliminary clinical experience with the Craniofacial-Plastic Surgical Robot in 6 genioplasty patients used filtering algorithms, adaptive control systems, and automated bone-drilling strategies to improve procedural stability [31]. The authors described the approach as safe and effective within this early cohort. However, the small sample size and first-in-human design mean that these findings should be interpreted as preliminary clinical feasibility rather than broad evidence of safety or effectiveness [31]. A robot-positioned cutting guide for fibular osteotomies in mandibular reconstruction was evaluated using 5 three-dimensional printed models and 1 cadaveric specimen. Fragment-length deviations measured 0.42 ± 0.29 mm in printed models and 1.00 ± 0.53 mm in the cadaveric model, while angular deviations reached 1.90 ± 1.22° and 1.94 ± 0.69°, respectively [32]. These results indicate low-millimeter technical accuracy in selected settings, but they do not establish broad clinical effectiveness because the evidence is still predominantly preclinical or limited to small first-in-human reports.

**Figure 2:**
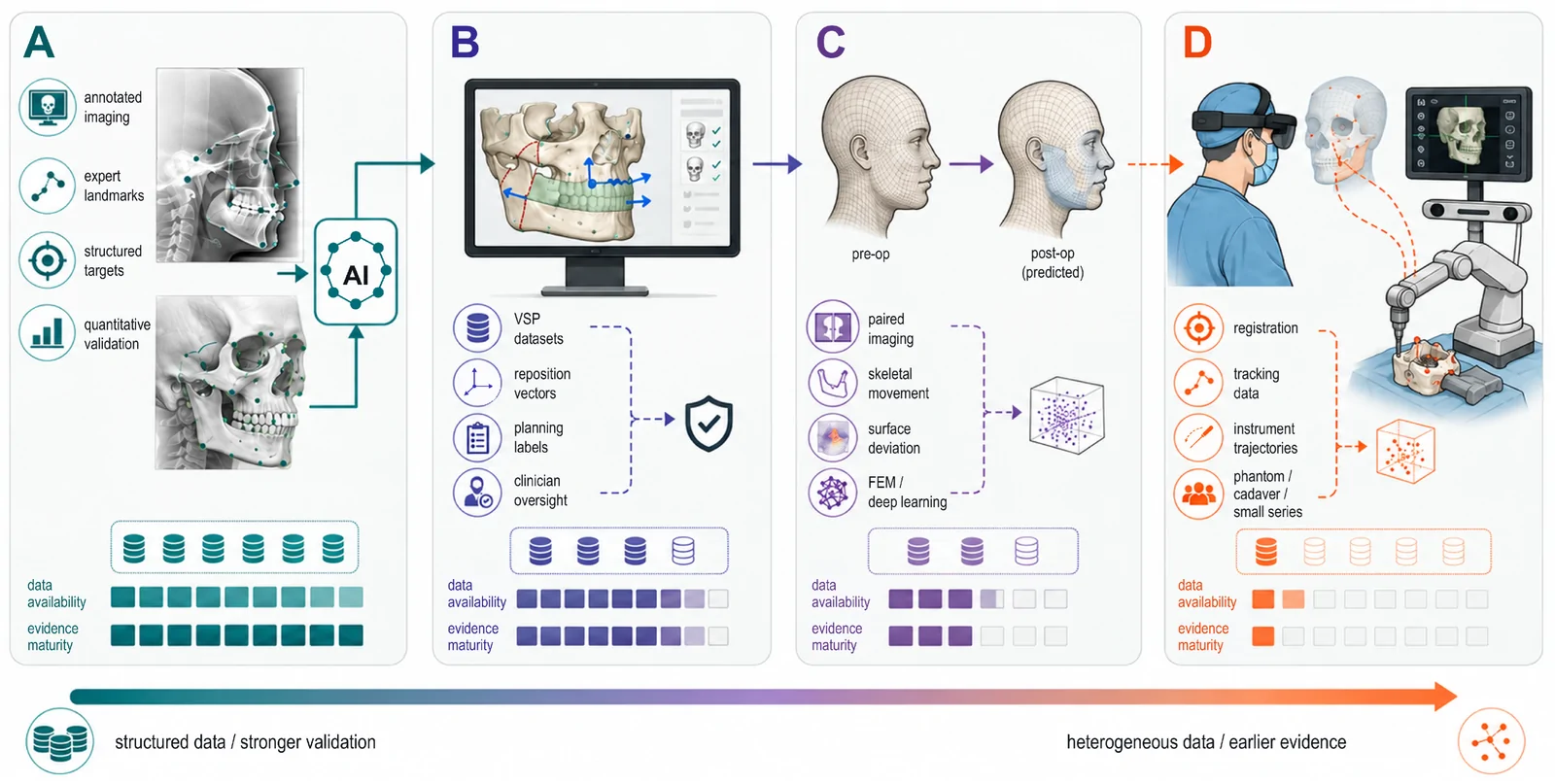
Data availability and evidence maturity across AI-enabled maxillofacial surgical tasks. Conceptual map of four clinical stages reviewed in orthognathic and reconstructive maxillofacial surgery. (**A**), Automated landmark detection is shown as the most data-structured domain, supported by annotated imaging, manual reference annotations, standardized anatomical targets, and quantitative validation. (**B**), AI-assisted diagnosis, osteotomy planning, and VSP are presented as an intermediate stage, supported by planning datasets, reposition vectors, planning labels, and clinician oversight. (**C**), Soft-tissue outcome prediction is shown as more data-limited because it depends on paired preoperative and postoperative imaging, skeletal-movement data, surface-deviation assessment, and FEM or deep learning models. (**D**), Intraoperative plan transfer is shown as the least standardized stage, relying on registration, tracking data, instrument trajectories, and evidence from phantom, cadaveric, or small clinical studies. Arrows and dashed connectors indicate conceptual workflow or information flow and do not encode effect size or certainty. Panel colors correspond to the lower left-to-right gradient from structured data and stronger validation to heterogeneous data and earlier evidence. Filled database icons and square cells qualitatively indicate relative data availability and evidence maturity. Their number is not a numerical score. Dots represent illustrative landmark, surface-deviation, or tracking and trajectory data. The lower gradient summarizes the review’s interpretation that evidence maturity tends to decrease as data become less structured, less standardized, and harder to validate across institutions. This figure should be interpreted as a conceptual synthesis of the reviewed literature, not as a formal grading system, causal proof, or definitive measure of clinical readiness [2,9,10,11,12,17,18,19,21,22,23,24,25,26,27,29,30,31,32,33,36,42,43,45,59,60,76,77,78]. Abbreviations: FEM, finite-element modeling; VSP, virtual surgical planning.

The difference between robotic accuracy and robotic clinical value should be emphasized. A robot can improve stability, repeatability, or guide positioning in a constrained technical task, but clinical value also depends on setup time, cost, sterilization workflow, safety protocols, intraoperative adaptability, surgeon control, haptic feedback, emergency override, and integration with VSP or navigation systems [76,77]. The broader robotics literature reinforces the need for cautious interpretation. The evolution of medical robotics from industrial robotic systems toward autonomous dental implant platforms illustrates that successful clinical deployment depends on cumulative engineering validation, workflow integration, safety assessment, and progressive clinical testing [76]. In orthognathic surgery, robotic systems integrated with VSP still face major barriers, including cost, infrastructure requirements, surgeon training, real-time feedback integration, and haptic technology [77]. These barriers are particularly relevant in craniomaxillofacial surgery because procedures are anatomically heterogeneous, highly patient-specific, and less standardized than implant-based workflows. A recent systematic review and meta-analysis of robotic surgical systems in oral and maxillofacial surgery reported promising accuracy and safety signals but rated the overall certainty of evidence as moderate to low, with most studies limited by small samples or short follow-up [78]. Figure 2 provides a conceptual summary of how data availability and evidence maturity vary across the four clinical stages reviewed, from structured image-based landmark detection to less standardized intraoperative plan-transfer technologies.

Intraoperative AR, navigation, and robotic-assisted execution represent an important but still early stage of the digital surgical pipeline. The available evidence supports technical feasibility and selected accuracy improvements in phantom, cadaveric, preclinical, and small clinical settings. It does not yet establish broad clinical effectiveness, routine superiority over conventional guides or navigation, or generalized safety across orthognathic and reconstructive indications. Their current role is best framed as support for plan transfer, visualization, registration, and selected execution tasks under surgeon supervision. Figure 2 summarizes the resulting gradient between data availability and evidence maturity across the four clinical stages reviewed, highlighting the more extensive quantitative validation for image-based landmarking, the emerging clinical validation of AI-assisted planning and VSP, the methodological but data-limited status of soft-tissue prediction, and the feasibility-dominant evidence base for intraoperative AR, navigation, and robotic-assisted plan transfer.

## 4. Discussion

This review shows that predictive AI and related data-driven surgical technologies are developing across the orthognathic and reconstructive maxillofacial pipeline, but not at the same pace or with the same evidentiary depth. Across the four clinical stages reviewed, the most consistent limitation is not simply model performance. It is the difficulty of generating, annotating, validating, and sharing the specific types of data required for each surgical task. This interpretation is consistent with broader surgical data science literature, which emphasizes that meaningful AI translation depends on perioperative data capture, structured annotation, validation, and integration into clinical workflows [3]. It is also consistent with broader medical AI literature, where clinical usefulness depends on external validation, prospective testing, and human-centered implementation rather than model architecture alone [79,80].

Quantitative validation is comparatively more developed where the task is anatomically defined, image-based, and supported by relatively structured datasets. Automated cephalometric and three-dimensional landmark detection benefits from common imaging modalities such as lateral cephalograms and CBCT, repeatable landmark definitions, and manual reference-annotation workflows. These conditions have allowed systematic reviews, meta-analyses, and multicenter validation studies to emerge. Even here, generalizability is not guaranteed. The distinction between public datasets and private institutional collections remains important, because models trained on restricted or single-center data may perform differently when applied to broader populations, different scanners, variable image quality, or more complex deformity patterns [2,11,12,13,21,34].

AI-assisted diagnosis, osteotomy planning, and VSP-related applications occupy a more complex evidentiary position. These models address tasks that are partly geometric but also clinical. Diagnosis, treatment objectives, surgeon preference, skeletal pattern, soft-tissue profile, and institutional planning philosophy all influence what counts as an acceptable output. Systematic reviews describe a small, heterogeneous, and largely retrospective evidence base that could not be pooled consistently [14,42]. Recent studies with larger datasets, prospective testing, and plan-comparison components are encouraging, but their outputs still require validation across surgeons, institutions, patient subgroups, and operative protocols. The current evidence therefore supports AI-assisted planning as a clinician-supervised adjunct to VSP rather than as an autonomous planning system. Future studies should specify whether the system is intended for diagnosis, plan generation, movement-vector prediction, visualization, or plan comparison because each task requires different validation criteria and carries a different level of clinical responsibility.

Soft-tissue prediction illustrates a different data problem. The task is clinically important because patients and surgeons often care as much about facial appearance and communication of expected outcomes as about skeletal movement alone. Yet the data needed to train and validate these models are harder to assemble than cephalometric landmarks. Reliable soft-tissue prediction requires paired preoperative and postoperative imaging, accurate surgical movement data, appropriate follow-up intervals, and enough biological variability to represent real patients. FEM-based models remain important because they incorporate biomechanical assumptions that purely data-driven approaches may miss, while deep learning models may reduce computation time and improve workflow efficiency in selected settings [25,26,27,59,60]. The main barrier is not a lack of promising algorithms. It is the limited availability of large, well-characterized, externally validated, longitudinal datasets [9,43,44]. This is also the domain where technical performance and patient-facing utility most clearly diverge: a simulation can be geometrically close to the postoperative scan but still fail to improve patient understanding, expectation management, or shared decision-making [56,62].

The evidence base for intraoperative AR navigation and robotic-assisted craniomaxillofacial surgery is less mature when judged by clinical effectiveness rather than technical accuracy alone. These technologies depend on registration, tracking, visualization, surgeon interaction, and procedural data that are difficult to capture consistently. The 33-case multicenter AR navigation series is an important clinical signal, but it was retrospective and heterogeneous rather than a prospective comparative trial [18]. Cadaveric, phantom, and preclinical studies are valuable for testing registration and execution accuracy, but they cannot by themselves establish superiority, safety, or patient benefit in live surgery [29,30,31,32,33]. This distinction is especially important in robotic-assisted CMF surgery, where the procedural landscape is more variable than in implant workflows. Orthognathic and reconstructive procedures often involve deformity-specific osteotomies, mobile skeletal segments, altered anatomy, and intraoperative adaptation, making direct extrapolation from implant robotics inappropriate [76,77]. Therefore, future AR and robotic studies should report not only overlay error or osteotomy deviation, but also workflow interruption, setup time, surgeon workload, intraoperative adaptability, complications, achieved skeletal position, and cost-related outcomes [8,78].

Viewed across the pipeline, the pattern is consistent: quantitative validation appears more developed where datasets are structured, accessible, and easier to annotate, and less mature where data are sparse, longitudinal, intraoperative, or highly procedure-specific. This relationship should be understood as an organizing interpretation of the literature rather than a causal conclusion proven by direct comparative testing. Annotated cephalometric and CBCT datasets have supported pooled estimates and external validation, whereas paired preoperative/postoperative datasets and intraoperative tracking data remain harder to standardize across institutions.

The clinical implication is that algorithmic accuracy should not be interpreted in isolation. A model with low geometric error in a controlled dataset may still perform unpredictably across imaging protocols, deformity patterns, surgical techniques, or institutional workflows. Likewise, successful phantom or cadaveric performance does not automatically translate into better patient outcomes. Future studies should therefore evaluate not only distance error, angular deviation, classification accuracy, or surface deviation, but also planning quality, decision impact, communication, operative efficiency, surgeon workload, safety, complications, cost-effectiveness, and patient-centered outcomes.

This shift in evaluation will require stronger data infrastructure. Multicenter prospective registries, standardized annotation protocols, geographically and demographically diverse imaging datasets, paired preoperative and postoperative scans, and curated intraoperative datasets would make model evaluation more clinically meaningful. Collaborative or federated data infrastructures may also help expand training and validation cohorts while addressing privacy and governance constraints [17,68,81]. The literature reviewed here suggests that the clinical translation of predictive AI and related digital surgical technologies in orthognathic and reconstructive maxillofacial surgery will depend on more than algorithmic sophistication. It will require the deliberate construction of high-quality, clinically annotated, longitudinally paired, intraoperatively relevant, and externally validated surgical data.

### 4.1. Evidence Gaps and Future Directions

The evidence reviewed across the surgical pipeline points to several recurring gaps that should shape future research. One of the most important is the limited availability of large, multicenter, externally validated datasets for orthognathic and reconstructive maxillofacial surgery. Landmark detection has advanced further in this regard because it often relies on structured imaging datasets and reproducible annotation workflows, with recent multicenter evaluations addressing some aspects of dataset heterogeneity and external testing [21,36]. By contrast, research on surgical planning, soft-tissue prediction, augmented-reality navigation, and robotic execution still depends heavily on single-center, retrospective, heterogeneous, or preclinical data [14,18,28,29,30,31,32,33,42,43,44]. This makes it difficult to determine whether reported performance reflects clinical generalizability or the characteristics of a local dataset [4,7].

Another major gap is the lack of longitudinal and paired data. Reliable soft-tissue prediction requires linked preoperative imaging, postoperative imaging, surgical-movement data, and standardized follow-up intervals. Without this structure, it is difficult to separate stable treatment effects from postoperative swelling, facial expression changes, orthodontic finishing, or measurement variability [9,43]. Similar longitudinal linkage is needed for planning and plan-transfer technologies. A virtual surgical plan becomes clinically meaningful only when it can be connected to achieved skeletal position, functional outcomes, complications, stability, and patient-reported benefit [45,62].

A further limitation is the absence of standardized evaluation metrics across studies. Depending on the technology and clinical task, studies may report mean radial error, surface deviation, classification accuracy, AUC, angular deviation, entry-point error, registration error, operative time, or qualitative acceptability [2,11]. Future research would benefit from task-specific core outcome sets that combine technical measures with clinically interpretable endpoints. Reports should also include dataset composition, annotation methods, external validation, subgroup performance, error analysis, and the intended clinical role of the technology [6,58].

Clinical and patient-centered outcomes also remain underrepresented. Most studies focus on geometric or technical endpoints, such as millimetric error, surface distance, overlay accuracy, or angular deviation. These measures are important for early validation, but they do not show whether a technology improves diagnosis, treatment planning, communication, operative efficiency, safety, satisfaction, function, esthetics, or long-term stability [4,5].

Prospective implementation studies are therefore needed. Much of the current evidence remains retrospective, methodological, cadaveric, phantom-based, or feasibility-oriented. These designs are appropriate during early development, but they are not sufficient to support routine clinical adoption [18,30]. Early stage clinical evaluation frameworks such as DECIDE-AI may help structure studies that examine human-AI interaction, workflow integration, safety signals, and decision impact before larger trials are conducted [8]. When these technologies are evaluated as clinical interventions, SPIRIT-AI and CONSORT-AI can also improve transparency and reproducibility [82,83].

Another important future direction is the development of collaborative data infrastructure. Multicenter registries, shared annotation protocols, harmonized virtual surgical planning data formats, and common reporting templates would make it easier to test models across imaging devices, patient populations, deformity patterns, surgical teams, and institutional workflows. Federated learning may help expand validation cohorts while preserving local data governance [84,85]. Privacy-preserving federated learning and uncertainty-aware medical imaging frameworks may be especially relevant for multi-institutional surgical imaging research [86,87].

Future studies should also distinguish more clearly between technical feasibility, clinical feasibility, clinical effectiveness, and implementation value. For example, a phantom study may show that an augmented-reality system reduces angular error under controlled conditions, but that does not prove that the system improves outcomes in live surgery [30]. Similarly, a cadaveric robotic study may demonstrate precise osteotomy execution, but it does not establish cost-effectiveness, safety, learning-curve behavior, or superiority over patient-specific guides [32,33].

Finally, clinician oversight should remain a central design principle. In orthognathic and reconstructive maxillofacial surgery, high-quality care depends on anatomy, occlusion, esthetics, function, stability, patient expectations, surgical feasibility, and intraoperative judgment. AI systems can support measurement, prediction, visualization, and plan transfer, but they should be evaluated by how well they augment clinical reasoning rather than by whether they replace it [4,5]. Explainability and interpretability are essential to this process, especially when clinicians must decide when to trust, override, or contextualize algorithmic output [88,89].

Future repositories should also follow principles that make data findable, accessible, interoperable, and reusable when this is ethically and legally possible [90]. For clinical AI systems intended for deployment, data infrastructure should be paired with traceability, usability, robustness, explainability, governance, and post-deployment monitoring [91]. Federated and privacy-preserving learning infrastructures may be particularly useful in maxillofacial surgery, where imaging and surgical data often cannot be centralized across institutions [85,86].

### 4.2. Limitations

This review has several limitations that should be considered when interpreting its conclusions. First, although the literature search was conducted in PubMed/MEDLINE, Embase, and Scopus using predefined database-specific strategies, the search was intended to support a structured narrative synthesis rather than a formal systematic or scoping review. Database-specific retrieval counts, duplicate-removal counts, screening counts, exclusion counts, and final-selection counts were not generated or retained. Relevant studies indexed exclusively in Web of Science, Cochrane Library, engineering databases, computer-science conference proceedings, or other specialty sources may have been missed. The search strategy and evidence-mapping framework are reported in the Supplementary Materials to improve transparency, but the review should not be interpreted as exhaustive across all biomedical, engineering, and computer-science databases. Second, the review was designed as a structured narrative synthesis rather than a systematic review, scoping review, or meta-analysis. PRISMA methodology was not applied, no formal risk-of-bias assessment was performed, and no quantitative pooling or certainty-of-evidence grading was undertaken. The conclusions therefore reflect a workflow-oriented interpretation of representative evidence, not pooled effect estimates or formal evidence certainty. Third, article selection was influenced by the scope of the review and by the accessibility of the available literature. Full-text articles were prioritized whenever available. When full text was unavailable, abstract-level information was used only when it contained sufficient detail to support the specific point cited. This approach may have limited the assessment of study design, validation methods, outcome definitions, and quantitative results in those cases. Fourth, the review intentionally focused on orthognathic and reconstructive maxillofacial surgery. Pediatric craniofacial, cleft-specific, temporomandibular joint, dental implant, and broader oral-surgery applications were discussed only when they informed the central surgical pipeline addressed in this article. The findings should therefore not be generalized to all AI applications in craniofacial surgery, oral surgery, implant dentistry, orthodontics, or dental digital workflows. Finally, the strength of the synthesis is constrained by the maturity of the underlying literature. Many studies were retrospective, single-center, technically oriented, or based on small clinical cohorts. Soft-tissue prediction studies frequently relied on limited paired preoperative and postoperative datasets, whereas intraoperative AR and robotic-assisted studies often involved case series, cadaveric models, preclinical validation, or three-dimensional printed phantoms. Outcome reporting was also heterogeneous. Most studies emphasized geometric or technical metrics, including mean radial error, distance error, surface deviation, classification accuracy, angular deviation, registration error, or feasibility. Fewer studies evaluated patient-centered outcomes, complication rates, operative time, cost-effectiveness, surgeon workload, clinical decision impact, or long-term clinical benefit. These limitations are especially relevant for intraoperative technologies, where low geometric error or successful registration does not necessarily translate into improved patient outcomes, lower complication rates, or better value of care [68,81]. For the same reason, the narrative evidence-maturity categories used in the review should be interpreted as descriptive and conceptual, not as formal grades equivalent to GRADE, PROBAST + AI, or other structured appraisal systems [37]. Evidence from broader healthcare AI, surgical data science, and reporting literature was used to support translational interpretation, but it should not be treated as direct evidence for effectiveness in every maxillofacial indication. Future updates of this review could use a formal systematic or scoping methodology, include additional engineering and computer-science databases, report full search and screening counts, apply formal appraisal tools, and separate evidence tables by study design, clinical directness, and validation level.

## 5. Conclusions

Predictive AI and related data-driven digital surgical technologies are now being investigated across several stages of orthognathic and reconstructive maxillofacial surgery, including imaging analysis, surgical planning, soft-tissue outcome prediction, and intraoperative plan transfer. The evidence, however, is uneven. Within the literature reviewed, automated cephalometric and three-dimensional landmark detection has undergone the most extensive quantitative evaluation, supported by systematic reviews, meta-analyses, and multicenter retrospective evaluations, including one study with independent external test sets. This does not imply high-certainty evidence or clinical readiness. Performance remains dependent on landmark type, imaging modality, dataset composition, annotation quality, and reference-standard construction.

AI-assisted diagnosis, bony planning, and osteotomy design show increasing technical capability, with studies reporting support for classification, reposition-vector prediction, and planning assistance. These findings are best interpreted as evidence for clinician-supervised decision support rather than autonomous surgical planning. Soft-tissue outcome prediction has also advanced through deep learning approaches alongside biomechanical FEM, but its clinical translation is constrained by the scarcity of paired preoperative and postoperative datasets, heterogeneous outcome definitions, and limited external validation. Intraoperative AR navigation and robotic-assisted craniomaxillofacial surgery provide promising evidence of plan-transfer feasibility in selected clinical, cadaveric, phantom, and preclinical settings, but current data do not establish broad clinical effectiveness or routine superiority over established workflows.

Across these domains, the maturity of clinical translation appears to be associated with the availability, structure, standardization, and external validation of the data required for each task. This should be understood as an organizing interpretation of the literature rather than a causal conclusion proven by direct comparative testing. Structured cephalometric and CBCT datasets have supported repeated quantitative synthesis and multicenter retrospective evaluation, whereas paired longitudinal imaging and intraoperative registration or tracking data remain harder to standardize and share. The differences observed across the pipeline do not appear to depend only on algorithmic sophistication. They also reflect whether each task has access to structured images, reproducible annotations, longitudinal paired data, VSP repositories, intraoperative tracking records, achieved-outcome measurements, and clinically meaningful endpoints.

For now, the most defensible role of predictive AI and related digital surgical technologies in orthognathic and reconstructive maxillofacial surgery is to support clinician-led planning, visualization, simulation, and plan transfer. Their clinical value will depend less on isolated performance metrics than on whether they can be validated across patients, institutions, imaging protocols, surgical workflows, and outcomes that matter clinically. In this sense, surgical judgment remains the central safeguard for responsible implementation, while better data infrastructure remains the condition for more reliable translation. Future research should therefore prioritize multicenter datasets, standardized metrics, external validation, prospective implementation studies, patient-centered outcomes, and collaborative or federated data infrastructure.

## Figures and Tables

**Table 1:** Representative reported metrics and study designs across clinical stages of predictive AI and related digital technologies in orthognathic and reconstructive maxillofacial surgery.

Clinical Stage/Application	Task	Representative Reported Metric	Sample Size/Evidence Base	Study Design	Interpretive Caution	Reference
Cephalometric AI, 2D	Automated landmark detection	Pooled mean radial error: 1.43 mm; 95% CI: 0.95–1.91 mm.	34 studies overall; 27 evaluated 2D landmarking; 14 studies contributing 21 estimates were included in the quantitative synthesis.	Systematic review and meta-analysis	Risk-of-bias concerns in patient selection and reference-standard construction, together with landmark-specific variation, limit clinical interpretation.	[11]
Cephalometric AI, 3D	Automated landmark detection	Pooled mean error: 2.44 mm; high heterogeneity reported	15 studies; 11 included in meta-analysis	Systematic review and meta-analysis	More heterogeneous than 2D landmarking because imaging protocols, landmark definitions, segmentation methods, and validation settings differ.	[12]
Cephalometric AI, 3D multicenter validation	Landmark detection using lightweight 3D U-Net	Mean radial error below 1.3 mm overall; below 1.4 mm in selected challenging subgroups	480 SCT and 240 CBCT scans for training; 320 SCT and 150 CBCT scans for inference	Multicenter retrospective diagnostic validation	More clinically informative than single-center development studies, but still dependent on dataset composition, annotation quality, and imaging modality.	[21]
AI-assisted osteotomy planning	Reposition-vector prediction using VSP transformer	Mean absolute error: 1.41 mm in validation and 1.34 mm in clinical testing	383 retrospective samples and 49 prospectively collected samples	Single-center model development with prospective testing	Supports planning-assistance potential, but broader external validation across institutions and surgical teams remains necessary.	[22]
ML-based planning decision support	Diagnostic classification and surgical planning support	Diagnostic success rate above 90% for six deformity categories; maxillary overdevelopment 89.27%; AUC above 0.88 for all diagnostic types	574 patients; surgical-plan comparison in 50-patient cohort	Single-center retrospective study with planning comparison	Should be interpreted as clinician-supervised decision support within a defined institutional dataset, not autonomous surgical planning.	[23]
Commercial AI cephalometric planning tools	Cephalometric measurements compared with 3D CT reference standard	Significant discrepancies reported for SNA, SNB, Wits appraisal, *Y*-axis angle, and facial-height ratios; ANB not significantly different	50 CT images from orthognathic surgery patients	Prospective comparative cohort	Findings apply to the evaluated tools and measurement categories and should not be generalized to all commercial AI systems.	[24]
Soft-tissue prediction, FEM	Postoperative facial surface prediction after double-jaw surgery	Overall surface deviation: 1.1 ± 0.3 mm; upper lip: 1.2 ± 0.7 mm; lower lip: 1.5 ± 0.7 mm; >80% qualitative acceptability reported	40 retrospective cases	Single-center retrospective validation	FEM remains a relevant biomechanical benchmark, but generalizability is limited by sample size, anatomy, surgical movement, and follow-up imaging.	[25]
Soft-tissue prediction, geometric DL	Facial shape-change prediction using FSC-Net	Accuracy comparable to a state-of-the-art FEM benchmark; 15-fold speed increase reported	Methodological validation using paired preoperative/postoperative data	Single-center methodological study	Supports workflow-efficiency potential, but does not establish clinical superiority over FEM or conventional planning tools.	[26]
Soft-tissue prediction, attention-based DL	Bone-to-soft-tissue movement correspondence using ACMT-Net	Accuracy reported as comparable to FEM, with improved computational efficiency	Methodological validation	Single-center methodological study	Evidence remains primarily methodological and dataset-dependent; external clinical validation is needed before routine use.	[27]
AR navigation, CMF/head and neck surgery	Intraoperative AR applications across trauma, orthognathic, tumor, and craniofacial cases	Feasibility of multiple AR-assisted intraoperative applications reported	33 cases	Multicenter retrospective case series	Demonstrates selected clinical use cases, but does not establish comparative effectiveness or broad procedural superiority.	[18]
AR navigation, Le Fort I osteotomy	Markerless AR-assisted maxillary positioning compared with VSP	SD of maxillary-position accuracy: 0.81 mm; 81.0% of measurements within 1 mm	6 patients	Single-center prospective clinical study	Clinically relevant but small; findings should be interpreted as early evidence for selected workflows.	[28]
AR navigation, zygomatic implants	Implant placement accuracy compared with surgical guides	Entry-point deviation: 2.43 ± 1.33 mm; 3D angular deviation: 5.80 ± 4.12°	5 cadaver skulls; 20 implants	Human cadaver validation study	Preclinical evidence; useful for technical validation but indirect for live surgical outcomes.	[29]
AR navigation, mandibular distraction	Distractor positioning compared with free-hand placement	61.6% reduction in angular error; 57% reduction in displacement error	40 distractors; 20 AR-guided and 20 free-hand	Phantom comparative study	Supports technical feasibility under experimental conditions; clinical transfer requires further validation.	[30]
Robot-assisted genioplasty	Preliminary clinical robotic-assisted execution	Preliminary safety and feasibility reported	6 patients	First-in-human single-center clinical report	Early clinical evidence from a very small cohort; not proof of broad safety or effectiveness.	[31]
Robot-assisted fibula osteotomy	Robot-positioned cutting guide compared with virtual plan	Cadaver fragment-length deviation: 1.00 ± 0.53 mm; angular deviation: 1.94 ± 0.69°	1 cadaveric specimen and 5 three-dimensional printed models	Preclinical validation study	Preclinical accuracy does not directly establish clinical benefit, workflow reliability, or patient-centered outcomes.	[32]
AR–robotic mandibular reconstruction	Combined AR navigation and robotic positioning	Mandibular osteotomy error: 1.47 ± 0.46 mm; fibular osteotomy error: 0.98 ± 0.24 mm	12 fibulas and 6 mandibles in cadaveric setting	Cadaveric validation study	Supports technical feasibility of combined AR–robotic workflows, but remains indirect evidence for clinical implementation.	[33]

## Data Availability

No new data were created or analyzed in this study. Data sharing is not applicable to this article.

## Supplementary Materials

The following supporting information can be downloaded at: https://www.mdpi.com/article/10.3390/dj14090605/s1. Table S1A: PubMed/MEDLINE search strategy framework and limits; Table S1B: Embase-adapted search strategy framework and limits; Table S1C: Scopus-adapted search strategy framework and limits; Table S2: eligibility and selection considerations; and Table S3: the evidence map used to structure the narrative synthesis.
