# Supplementary material for: Artificial Intelligence and Digital Technologies in Orthognathic and Reconstructive Maxillofacial Surgery: Data Availability and Evidence Maturity
Source: Dent J (Basel). 2026 Sep 18;14(9):605. doi: 10.3390/dj14090605 (PMC13605402; doi:10.3390/dj14090605)
Supplement: Supplementary file 1 [file dentistry-14-00605-s001.zip › dentistry-4513449-supplementary.pdf]

Supplementary Material

Artificial Intelligence and Digital Technologies in Orthognathic and Reconstructive Maxillofacial Surgery: Data Availability and Evidence Maturity

Purpose

This supplementary material documents the literature search strategies used in PubMed/MEDLINE, Embase, and Scopus, the domain-specific search frameworks, eligibility criteria, selection considerations, and evidence map used to support the structured narrative review. Database results were reviewed manually by two authors (M.C.-D. and J.M.P.-V.), and no automated tools were used for source selection. The supplement is intended to improve transparency for a workflow-oriented narrative synthesis. It should not be interpreted as a PRISMA flow record, formal systematic review protocol, scoping review protocol, meta-analysis dataset, or risk-of-bias assessment.

The full search framework is reported here rather than in the main Methods section to preserve readability while documenting the logic used to identify literature across the clinical pipeline of orthognathic and reconstructive maxillofacial surgery. PubMed/MEDLINE was used to develop the base search framework, and the search syntax was adapted for Embase and Scopus. Because this review was designed as a narrative review, source identification and selection were iterative and did not follow a formal title/abstract and full-text screening sequence. A PRISMA-style screening record was therefore not maintained. Database-specific retrieval counts, duplicate-removal counts, screening counts, exclusion counts, and final-selection counts were not generated or retained. Instead, predefined database-specific search strategies were used to identify relevant evidence and build a conceptual evidence map linking each stage of the surgical workflow to the data types, validation strategies, and evidence characteristics available for that stage. This approach allowed the review to compare evidence maturity across domains without implying that numerical accuracy metrics from different tasks, imaging modalities, and validation settings are directly interchangeable.

Supplementary Table S1A. PubMed/MEDLINE search strategy framework and limits

Search ID	Purpose	Database	Date range	Limits applied	Exact PubMed/MEDLINE query framework
S1	Primary search for orthognathic and reconstructive maxillofacial surgery, AI, machine learning, deep learning, virtual surgical planning, computer-assisted surgery, navigation, augmented reality, and robotics.	PubMed/MEDLINE	January 15, 2018 to April 15, 2026	English-language, peer-reviewed biomedical literature; selected pre-2018 foundational studies were considered when directly relevant.	(("Orthognathic Surgery"[Mesh] OR orthognathic[tiab] OR "dentofacial deformity"[tiab] OR "maxillofacial surgery"[tiab] OR "craniomaxillofacial"[tiab] OR "craniofacial surgery"[tiab] OR "mandibular reconstruction"[tiab] OR "maxillary reconstruction"[tiab] OR "facial skeleton"[tiab]) AND ("Artificial Intelligence"[Mesh] OR "Machine Learning"[Mesh] OR "Deep Learning"[Mesh] OR "Neural Networks, Computer"[Mesh] OR "Computer-Assisted Surgery"[tiab] OR "Surgery, Computer-Assisted"[Mesh] OR "virtual surgical planning"[tiab] OR VSP[tiab] OR "computer-assisted surgery"[tiab] OR "computer assisted surgery"[tiab] OR "augmented reality"[tiab] OR "mixed reality"[tiab] OR "surgical

					navigation"[tiab] OR "robotic surgery"[tiab] OR "robot-assisted"[tiab] OR "robot assisted"[tiab]))
S2	Cephalometric AI, CBCT, CT, automated landmark detection, and three-dimensional landmarking.	PubMed/MEDLINE	January 15, 2018 to April 15, 2026	English-language, peer-reviewed biomedical literature.	(("Cephalometry"[Mesh] OR cephalometric[tiab] OR cephalometry[tiab] OR "lateral cephalogram"[tiab] OR CBCT[tiab] OR "cone-beam computed tomography"[tiab] OR "cone beam computed tomography"[tiab] OR "computed tomography"[tiab] OR "3D cephalometric"[tiab] OR "three-dimensional cephalometric"[tiab]) AND ("Artificial Intelligence"[Mesh] OR "Machine Learning"[Mesh] OR "Deep Learning"[Mesh] OR "Neural Networks, Computer"[Mesh] OR "automated landmark"[tiab] OR "landmark detection"[tiab] OR "landmark identification"[tiab] OR "landmark localization"[tiab] OR "deep learning"[tiab] OR "convolutional neural network"[tiab] OR CNN[tiab]))
S3	AI-assisted diagnosis, osteotomy planning, reposition-vector prediction, surgical need prediction, and virtual surgical planning.	PubMed/MEDLINE	January 15, 2018 to April 15, 2026	English-language, peer-reviewed biomedical literature.	((orthognathic[tiab] OR "dentofacial deformity"[tiab] OR "maxillofacial surgery"[tiab] OR "craniomaxillofacial"[tiab]) AND ("virtual surgical planning"[tiab] OR VSP[tiab] OR "osteotomy planning"[tiab] OR "surgical planning"[tiab] OR "treatment planning"[tiab] OR "surgical need"[tiab] OR "decision support"[tiab] OR "diagnosis"[tiab] OR "reposition vector"[tiab]) AND ("Artificial Intelligence"[Mesh] OR "Machine Learning"[Mesh] OR "Deep Learning"[Mesh] OR "neural network"[tiab] OR transformer[tiab] OR "generative model"[tiab] OR "diffusion model"[tiab]))
S4	Soft-tissue outcome prediction, postoperative facial simulation, FEM, biomechanical modeling, point-cloud models, and deep learning.	PubMed/MEDLINE	January 15, 2018 to April 15, 2026	English-language, peer-reviewed biomedical literature; selected pre-2018 foundational FEM work was considered when directly relevant.	((orthognathic[tiab] OR "double-jaw surgery"[tiab] OR "maxillofacial surgery"[tiab]) AND ("soft tissue"[tiab] OR "facial soft tissue"[tiab] OR "facial appearance"[tiab] OR "postoperative facial"[tiab] OR "facial morphology"[tiab] OR "outcome prediction"[tiab] OR simulation[tiab]) AND ("finite element"[tiab] OR FEM[tiab] OR biomechanical[tiab] OR "mass spring"[tiab] OR "deep learning"[tiab] OR "point cloud"[tiab] OR PointNet[tiab] OR transformer[tiab] OR "machine learning"[tiab] OR "Artificial Intelligence"[Mesh]))
S5	Intraoperative navigation, augmented reality, mixed reality, registration, robotic assistance,	PubMed/MEDLINE	January 15, 2018 to April 15, 2026	English-language, peer-reviewed biomedical literature.	(("maxillofacial surgery"[tiab] OR "craniomaxillofacial"[tiab] OR "craniofacial surgery"[tiab] OR orthognathic[tiab] OR

	and plan transfer in CMF or reconstructive surgery.				"mandibular reconstruction"[tiab] OR "fibula flap"[tiab] OR "head and neck surgery"[tiab]) AND ("augmented reality"[tiab] OR "mixed reality"[tiab] OR "surgical navigation"[tiab] OR navigation[tiab] OR registration[tiab] OR "image-to-patient registration"[tiab] OR tracking[tiab] OR "robot-assisted"[tiab] OR "robot assisted"[tiab] OR robotics[tiab] OR "Robotics"[Mesh] OR "Surgery, Computer-Assisted"[Mesh]))
S6	Clinical translation, validation, generalizability, bias, regulation, implementation, data governance, and human oversight for AI-assisted surgical technologies.	PubMed/MEDLINE	January 15, 2018 to April 15, 2026	English-language, peer-reviewed biomedical literature.	(("Artificial Intelligence"[Mesh] OR "Machine Learning"[Mesh] OR "Deep Learning"[Mesh] OR "Surgery, Computer-Assisted"[Mesh] OR "surgical data science"[tiab] OR "AI-assisted"[tiab] OR "AI-based"[tiab]) AND (surgery[tiab] OR surgical[tiab] OR maxillofacial[tiab] OR craniomaxillofacial[tiab] OR orthognathic[tiab]) AND (validation[tiab] OR "external validation"[tiab] OR generalizability[tiab] OR bias[tiab] OR "risk of bias"[tiab] OR implementation[tiab] OR regulation[tiab] OR governance[tiab] OR transparency[tiab] OR accountability[tiab] OR "human oversight"[tiab] OR "patient safety"[tiab]))

Supplementary Table S1B. Embase-adapted search strategy framework and limits

Search ID	Purpose	Database	Date range	Limits applied	Embase-adapted query framework
E1	Primary search for orthognathic and reconstructive maxillofacial surgery, AI, machine learning, deep learning, virtual surgical planning, computer-assisted surgery, navigation, augmented reality, and robotics.	Embase	January 15, 2018 to April 15, 2026	English-language, peer-reviewed biomedical literature; selected pre-2018 foundational studies were considered when directly relevant.	(('orthognathic surgery'/exp OR orthognathic:ti,ab,kw OR 'dentofacial deformity':ti,ab,kw OR 'maxillofacial surgery':ti,ab,kw OR craniomaxillofacial:ti,ab,kw OR 'craniofacial surgery':ti,ab,kw OR 'mandibular reconstruction':ti,ab,kw OR 'maxillary reconstruction':ti,ab,kw OR 'facial skeleton':ti,ab,kw) AND ('artificial intelligence'/exp OR 'machine learning'/exp OR 'deep learning'/exp OR 'neural network'/exp OR 'computer assisted surgery'/exp OR 'computer-assisted surgery':ti,ab,kw OR 'virtual surgical planning':ti,ab,kw OR VSP:ti,ab,kw OR 'computer-assisted surgery':ti,ab,kw OR 'computer assisted surgery':ti,ab,kw OR 'augmented reality':ti,ab,kw OR 'mixed reality':ti,ab,kw OR 'surgical navigation':ti,ab,kw OR 'robotic surgery':ti,ab,kw OR 'robot-assisted':ti,ab,kw OR 'robot assisted':ti,ab,kw))

E2	Cephalometric AI, CBCT, CT, automated landmark detection, and three-dimensional landmarking.	Embase	January 15, 2018 to April 15, 2026	English-language, peer-reviewed biomedical literature.	(('cephalometry'/exp OR cephalometric:ti,ab,kw OR cephalometry:ti,ab,kw OR 'lateral cephalogram':ti,ab,kw OR CBCT:ti,ab,kw OR 'cone-beam computed tomography':ti,ab,kw OR 'cone beam computed tomography':ti,ab,kw OR 'computed tomography':ti,ab,kw OR '3D cephalometric':ti,ab,kw OR 'three-dimensional cephalometric':ti,ab,kw) AND ('artificial intelligence'/exp OR 'machine learning'/exp OR 'deep learning'/exp OR 'neural network'/exp OR 'automated landmark':ti,ab,kw OR 'landmark detection':ti,ab,kw OR 'landmark identification':ti,ab,kw OR 'landmark localization':ti,ab,kw OR 'deep learning':ti,ab,kw OR 'convolutional neural network':ti,ab,kw OR CNN:ti,ab,kw))
E3	AI-assisted diagnosis, osteotomy planning, reposition-vector prediction, surgical need prediction, and virtual surgical planning.	Embase	January 15, 2018 to April 15, 2026	English-language, peer-reviewed biomedical literature.	((orthognathic:ti,ab,kw OR 'dentofacial deformity':ti,ab,kw OR 'maxillofacial surgery':ti,ab,kw OR craniomaxillofacial:ti,ab,kw) AND ('virtual surgical planning':ti,ab,kw OR VSP:ti,ab,kw OR 'osteotomy planning':ti,ab,kw OR 'surgical planning':ti,ab,kw OR 'treatment planning':ti,ab,kw OR 'surgical need':ti,ab,kw OR 'decision support':ti,ab,kw OR diagnosis:ti,ab,kw OR 'reposition vector':ti,ab,kw) AND ('artificial intelligence'/exp OR 'machine learning'/exp OR 'deep learning'/exp OR 'neural network':ti,ab,kw OR transformer:ti,ab,kw OR 'generative model':ti,ab,kw OR 'diffusion model':ti,ab,kw))
E4	Soft-tissue outcome prediction, postoperative facial simulation, FEM, biomechanical modeling, point-cloud models, and deep learning.	Embase	January 15, 2018 to April 15, 2026	English-language, peer-reviewed biomedical literature; selected pre-2018 foundational FEM work was considered when directly relevant.	((orthognathic:ti,ab,kw OR 'double-jaw surgery':ti,ab,kw OR 'maxillofacial surgery':ti,ab,kw) AND ('soft tissue':ti,ab,kw OR 'facial soft tissue':ti,ab,kw OR 'facial appearance':ti,ab,kw OR 'postoperative facial':ti,ab,kw OR 'facial morphology':ti,ab,kw OR 'outcome prediction':ti,ab,kw OR simulation:ti,ab,kw) AND ('finite element':ti,ab,kw OR FEM:ti,ab,kw OR biomechanical:ti,ab,kw OR 'mass spring':ti,ab,kw OR 'deep learning':ti,ab,kw OR 'point cloud':ti,ab,kw OR PointNet:ti,ab,kw OR transformer:ti,ab,kw OR 'machine learning':ti,ab,kw OR 'artificial intelligence'/exp))
E5	Intraoperative navigation, augmented reality, mixed reality, registration, robotic assistance, and plan transfer in CMF or reconstructive surgery.	Embase	January 15, 2018 to April 15, 2026	English-language, peer-reviewed biomedical literature.	(('maxillofacial surgery':ti,ab,kw OR craniomaxillofacial:ti,ab,kw OR 'craniofacial surgery':ti,ab,kw OR orthognathic:ti,ab,kw OR 'mandibular reconstruction':ti,ab,kw OR 'fibula

					flap':ti,ab,kw OR 'head and neck surgery':ti,ab,kw) AND ('augmented reality':ti,ab,kw OR 'mixed reality':ti,ab,kw OR 'surgical navigation':ti,ab,kw OR navigation:ti,ab,kw OR registration:ti,ab,kw OR 'image-to-patient registration':ti,ab,kw OR tracking:ti,ab,kw OR 'robot-assisted':ti,ab,kw OR 'robot assisted':ti,ab,kw OR robotics:ti,ab,kw OR 'robotics'/exp OR 'computer assisted surgery'/exp))
E6	Clinical translation, validation, generalizability, bias, regulation, implementation, data governance, and human oversight for AI-assisted surgical technologies.	Embase	January 15, 2018 to April 15, 2026	English-language, peer-reviewed biomedical literature.	(('artificial intelligence'/exp OR 'machine learning'/exp OR 'deep learning'/exp OR 'computer assisted surgery'/exp OR 'surgical data science':ti,ab,kw OR 'AI-assisted':ti,ab,kw OR 'AI-based':ti,ab,kw) AND (surgery:ti,ab,kw OR surgical:ti,ab,kw OR maxillofacial:ti,ab,kw OR craniomaxillofacial:ti,ab,kw OR orthognathic:ti,ab,kw) AND (validation:ti,ab,kw OR 'external validation':ti,ab,kw OR generalizability:ti,ab,kw OR bias:ti,ab,kw OR 'risk of bias':ti,ab,kw OR implementation:ti,ab,kw OR regulation:ti,ab,kw OR governance:ti,ab,kw OR transparency:ti,ab,kw OR accountability:ti,ab,kw OR 'human oversight':ti,ab,kw OR 'patient safety':ti,ab,kw))

Supplementary Table S1C. Scopus-adapted search strategy framework and limits

Search ID	Purpose	Database	Date range	Limits applied	Scopus-adapted query framework
SC1	Primary search for orthognathic and reconstructive maxillofacial surgery, AI, machine learning, deep learning, virtual surgical planning, computer-assisted surgery, navigation, augmented reality, and robotics.	Scopus	January 15, 2018 to April 15, 2026	English-language, peer-reviewed biomedical literature; selected pre-2018 foundational studies were considered when directly relevant.	TITLE-ABS-KEY(("orthognathic surgery" OR orthognathic OR "dentofacial deformity" OR "maxillofacial surgery" OR craniomaxillofacial OR "craniofacial surgery" OR "mandibular reconstruction" OR "maxillary reconstruction" OR "facial skeleton") AND ("artificial intelligence" OR "machine learning" OR "deep learning" OR "neural network" OR "computer-assisted surgery" OR "computer assisted surgery" OR "virtual surgical planning" OR VSP OR "augmented reality" OR "mixed reality" OR "surgical navigation" OR "robotic surgery" OR "robot-assisted" OR "robot assisted"))
SC2	Cephalometric AI, CBCT, CT, automated landmark detection, and three-dimensional landmarking.	Scopus	January 15, 2018 to April 15, 2026	English-language, peer-reviewed biomedical literature.	TITLE-ABS-KEY((cephalometric OR cephalometry OR "lateral cephalogram" OR CBCT OR "cone-beam computed tomography" OR "cone beam computed tomography" OR "computed tomography" OR "3D cephalometric" OR "three-dimensional

					cephalometric") AND ("artificial intelligence" OR "machine learning" OR "deep learning" OR "neural network" OR "automated landmark" OR "landmark detection" OR "landmark identification" OR "landmark localization" OR "convolutional neural network" OR CNN))
SC3	AI-assisted diagnosis, osteotomy planning, reposition-vector prediction, surgical need prediction, and virtual surgical planning.	Scopus	January 15, 2018 to April 15, 2026	English-language, peer-reviewed biomedical literature.	TITLE-ABS-KEY((orthognathic OR "dentofacial deformity" OR "maxillofacial surgery" OR craniomaxillofacial) AND ("virtual surgical planning" OR VSP OR "osteotomy planning" OR "surgical planning" OR "treatment planning" OR "surgical need" OR "decision support" OR diagnosis OR "reposition vector") AND ("artificial intelligence" OR "machine learning" OR "deep learning" OR "neural network" OR transformer OR "generative model" OR "diffusion model"))
SC4	Soft-tissue outcome prediction, postoperative facial simulation, FEM, biomechanical modeling, point-cloud models, and deep learning.	Scopus	January 15, 2018 to April 15, 2026	English-language, peer-reviewed biomedical literature; selected pre-2018 foundational FEM work was considered when directly relevant.	TITLE-ABS-KEY((orthognathic OR "double-jaw surgery" OR "maxillofacial surgery") AND ("soft tissue" OR "facial soft tissue" OR "facial appearance" OR "postoperative facial" OR "facial morphology" OR "outcome prediction" OR simulation) AND ("finite element" OR FEM OR biomechanical OR "mass spring" OR "deep learning" OR "point cloud" OR PointNet OR transformer OR "machine learning" OR "artificial intelligence"))
SC5	Intraoperative navigation, augmented reality, mixed reality, registration, robotic assistance, and plan transfer in CMF or reconstructive surgery.	Scopus	January 15, 2018 to April 15, 2026	English-language, peer-reviewed biomedical literature.	TITLE-ABS-KEY(("maxillofacial surgery" OR craniomaxillofacial OR "craniofacial surgery" OR orthognathic OR "mandibular reconstruction" OR "fibula flap" OR "head and neck surgery") AND ("augmented reality" OR "mixed reality" OR "surgical navigation" OR navigation OR registration OR "image-to-patient registration" OR tracking OR "robot-assisted" OR "robot assisted" OR robotics))
SC6	Clinical translation, validation, generalizability, bias, regulation, implementation, data governance, and human oversight for AI-assisted surgical technologies.	Scopus	January 15, 2018 to April 15, 2026	English-language, peer-reviewed biomedical literature.	TITLE-ABS-KEY(("artificial intelligence" OR "machine learning" OR "deep learning" OR "computer-assisted surgery" OR "computer assisted surgery" OR "surgical data science" OR "AI-assisted" OR "AI-based") AND (surgery OR surgical OR maxillofacial OR craniomaxillofacial OR orthognathic) AND (validation OR "external validation" OR generalizability OR bias OR "risk of bias" OR implementation OR regulation OR governance OR transparency OR accountability OR "human oversight" OR "patient safety"))

Note: The PubMed/MEDLINE strategy was the base framework and was adapted to the vocabulary and field structure of Embase and the title/abstract/keyword structure of Scopus. Search results informed the structured narrative synthesis. Because source selection was iterative, database-specific retrieval, duplicate-removal, screening, exclusion, and final-selection counts were not generated or retained. No PRISMA-style screening sequence or formal risk-of-bias assessment was performed.

Abbreviations: AI, artificial intelligence; AR, augmented reality; CBCT, cone-beam computed tomography; CMF, craniomaxillofacial; CT, computed tomography; EMTREE, Embase subject headings; FEM, finite-element method; MeSH, Medical Subject Headings; VSP, virtual surgical planning; tiab, title/abstract field tag; ti,ab,kw, title/abstract/keyword fields; TITLE-ABS-KEY, Scopus title/abstract/keyword field search.

Supplementary Table S2. Eligibility and selection considerations

Rationale for four-stage synthesis structure: the four stages correspond to the main points at which data-driven technologies interact with the surgical workflow: anatomical quantification, diagnostic and surgical planning, postoperative outcome simulation, and intraoperative plan transfer. These stages also differ in data availability: landmark detection is supported by structured imaging and annotation workflows; planning models depend on institutional surgical decisions and VSP data; soft-tissue prediction requires paired longitudinal imaging; and intraoperative guidance or robotic execution requires registration, tracking, calibration, procedural, and achieved-outcome data.
Organization after selection: Selected sources were assigned to one or more workflow domains: cephalometric and three-dimensional landmark detection, AI-assisted diagnosis, osteotomy planning and VSP, soft-tissue outcome prediction, intraoperative navigation, AR and robotic-assisted plan transfer, or clinical translation and validation. Each source was mapped by its dominant data type and described according to study design, validation setting, dataset and sample characteristics, reference standard or comparator, reported technical or clinical outcomes, and clinical directness.

**Item**	**Specification used in the review**
Information source	PubMed/MEDLINE, Embase, and Scopus. Reference lists of relevant systematic reviews, meta-analyses, scoping reviews, umbrella reviews, narrative reviews, and key clinical or technical studies were checked when they clarified a technology, workflow, or validation issue.
Publication-date range	January 15, 2018 to April 15, 2026. Selected pre-2018 foundational studies were considered when directly relevant to FEM, soft-tissue prediction, surgical navigation, or computer-assisted maxillofacial surgery.
Eligibility criteria	Sources were eligible when they addressed one or more of the following domains: cephalometric AI and three-dimensional landmark detection, AI-assisted diagnosis, osteotomy planning, virtual surgical planning, soft-tissue outcome prediction, intraoperative navigation, augmented reality, robotic-assisted craniomaxillofacial surgery, or clinical translation, validation, data infrastructure, and human oversight.
Eligible evidence types	Systematic reviews, meta-analyses, scoping reviews, umbrella reviews, narrative reviews, original clinical studies, retrospective and prospective cohorts, randomized evidence when available, technical validation studies, cadaveric studies, phantom studies, first-in-human reports, and translational reports.

Item	Specification used in the review
Selection considerations	Selection of representative sources was guided by the availability of domain-level synthesis and by reported external or prospective validation, comparison with a relevant reference standard or surgical plan, quantitative performance or clinical outcome data, intraoperative feasibility, or implementation barriers. Systematic reviews and meta-analyses were prioritized when they provided relevant domain-level synthesis.
Exclusion criteria	Sources were not selected for detailed discussion when they primarily addressed administrative AI, nonsurgical workflow optimization, voice-based documentation, triage chatbots, or dental implant planning without direct relevance to reconstructive or craniomaxillofacial surgical workflows. Sources were also not emphasized when they lacked sufficient methodological or outcome detail for the cited point or when another source addressed that point more directly.
Synthesis approach	Workflow-oriented narrative synthesis organized by clinical stage. Evidence-maturity judgments were interpretive and were not derived from a numerical score or formal certainty grade. No formal PRISMA flow diagram, risk-of-bias assessment, quantitative pooling, or database-specific record-count reporting was performed.
Review classification	Structured narrative review. The review used predefined database-specific search strategies, eligibility criteria, selection considerations, and evidence mapping, but it was not designed as a formal systematic review, scoping review, or meta-analysis.
Interpretation of numerical estimates	Numerical estimates were interpreted within their original study context. Cross-domain comparisons were treated as indirect because studies differed in task, modality, population, validation design, comparator, metric, and clinical indication.
Organization after selection	Selected sources were assigned to one or more workflow domains and mapped by dominant data type. They were then described according to study design, validation setting, dataset and sample characteristics, reference standard or comparator, reported technical or clinical outcomes, and clinical directness.
Rationale for four-stage synthesis structure	The four stages reflect sequential data environments in the surgical workflow: anatomical quantification, diagnostic/planning support, postoperative simulation, and intraoperative plan transfer. Each stage has different data availability, standardization, and validation constraints.

Supplementary Table S3. Evidence map used to structure the narrative synthesis

The evidence map was constructed to support a narrative comparison of evidence maturity across workflow stages. Evidence maturity was interpreted according to the dominant evidence base, clinical directness, validation setting, and data requirements of each technology. These judgments were interpretive and were not derived from a numerical score or formal certainty grade.

Evidence block	Manuscript reference group	Dominant evidence base	Main outcome or evidence characteristic	Clinical directness	Role in the review	Cautions for interpretation
Cephalometric AI and 2D landmark detection	[11,13,34,35]	Systematic reviews, meta-analysis, umbrella review, and narrative/systematic review literature	Mean radial error, proportion of landmarks within tolerance thresholds, time savings, landmark-specific variability	Direct for landmarking accuracy; indirect for final surgical outcomes	Establishes the most quantitatively developed imaging-based AI domain in the review	Accuracy varies by landmark, image quality, annotation protocol, population, and reference standard.
Three-dimensional landmarking and CBCT/CT validation	[11,12,21,36]	Systematic reviews, meta-analysis, and multicenter retrospective diagnostic validation	3D landmarking error, heterogeneity, external validation across CT/CBCT datasets	Direct for 3D landmarking performance; indirect for treatment planning quality	Supports the interpretation that structured imaging datasets can facilitate repeated quantitative evaluation	3D performance is more heterogeneous than 2D performance and remains modality- and dataset-dependent.
AI-assisted diagnosis and osteotomy/VSP planning	[2,10,14–16,22–24,42,45–54]	Systematic/scoping reviews, model-development studies, prospective testing, comparative cohorts, and bibliometric analysis	Diagnostic classification, surgical-need prediction, reposition-vector prediction, plan comparison, VSP workflow evidence	Direct for model performance and planning agreement; less direct for patient-centered outcomes	Frames AI planning as clinician-supervised decision support rather than autonomous surgical planning	Models may reflect institutional planning preferences and require validation across surgeons, populations, and workflows.
Commercial AI cephalometric planning tools	[24]	Prospective comparative cohort	Agreement and discrepancy between commercial AI measurements and 3D CT reference standards	Direct for evaluated measurements and tools; indirect for overall commercial AI reliability	Provides caution against equating commercial availability with validated clinical accuracy	Findings apply to the evaluated tools, measurements, and patient group only.
Soft-tissue prediction: FEM and biomechanical modeling	[25,59–61]	Retrospective validation, probabilistic FEM, incremental FEM, and small prospective multimodal study	Surface deviation, lip-region prediction, uncertainty modeling, anatomical-detail integration	Direct for soft-tissue simulation accuracy in selected cohorts; indirect for patient	Preserves FEM as a biomechanical benchmark and not merely a historical comparator	Small cohorts, anatomical variability, follow-up timing, and imaging protocols limit generalizability.

Evidence block	Manuscript reference group	Dominant evidence base	Main outcome or evidence characteristic	Clinical directness	Role in the review	Cautions for interpretation
				satisfaction and decision impact		
Soft-tissue prediction: deep learning and point-cloud models	[26,27,43,57,63–66]	Methodological validation studies, proof-of-concept studies, scoping/systematic review evidence	Computational efficiency, geometric correspondence, facial shape-change prediction, model feasibility	Direct for technical simulation performance; indirect for clinical decision-making and outcomes	Shows how deep learning may reduce computational burden and support planning visualization	External validation, paired longitudinal imaging, and standardized outcome reporting remain limited.
AR navigation and intraoperative plan transfer	[18,19,28–30,67,69–75]	Systematic review of registration, retrospective case series, small prospective studies, cadaveric studies, phantom studies, and pilot evaluations	Registration strategy, overlay accuracy, positional deviation, angular error, feasibility, workflow integration	Direct for technical accuracy and feasibility; variable for clinical effectiveness	Positions AR as a plan-transfer and visualization technology rather than purely predictive AI	Evidence is heterogeneous and often early-stage; phantom/cadaveric performance does not establish clinical superiority.
Robotic-assisted craniomaxillofacial surgery	[31–33,76–78]	First-in-human report, preclinical validation, cadaveric validation, and contextual robotics reviews	Technical execution, osteotomy deviation, robotic positioning, workflow barriers	Direct for selected technical accuracy metrics; limited for general clinical effectiveness	Frames robotics as an emerging execution-support technology requiring careful translational calibration	Evidence is platform-specific and limited by small cohorts, preclinical settings, cost, training, and workflow complexity.
Clinical translation, validation, data infrastructure, and human oversight	[17,68,81]	Critical review, surgical data science literature, and broad AI decision-making review	Dataset bias, external validation, governance, clinician oversight, patient-centered outcomes, infrastructure needs	Conceptually direct for implementation; indirect for technology-specific performance	Provides the cross-cutting framework for responsible translation across the pipeline	General surgical AI literature should not be overextended as direct evidence for every maxillofacial indication.
